# Functional Neural Correlates of Anosognosia in Mild Cognitive Impairment and Alzheimer’s Disease: a Systematic Review

**DOI:** 10.1007/s11065-019-09410-x

**Published:** 2019-06-03

**Authors:** Jaime D. Mondragón, Natasha M. Maurits, Peter P. De Deyn

**Affiliations:** 1 Department of Neurology, University of Groningen, University Medical Center Groningen, PO Box 30001, 9700 RB Groningen, the Netherlands; 2 Alzheimer Research Center, University of Groningen, University Medical Center Groningen, Groningen, The Netherlands; 3 Institute Born-Bunge, Laboratory of Neurochemistry and Behavior, University of Antwerp, Antwerp, Belgium

**Keywords:** Alzheimer, Anosognosia, Connectivity, Metabolism, Perfusion, Mild cognitive impairment

## Abstract

Functional neuroimaging techniques (i.e. single photon emission computed tomography, positron emission tomography, and functional magnetic resonance imaging) have been used to assess the neural correlates of anosognosia in mild cognitive impairment (MCI) and Alzheimer’s disease (AD). A systematic review of this literature was performed, following the Preferred Reporting Items for Systematic Reviews and Meta Analyses statement, on PubMed, EMBASE, and PsycINFO databases. Twenty-five articles met all inclusion criteria. Specifically, four brain connectivity and 21 brain perfusion, metabolism, and activation articles. Anosognosia is associated in MCI with frontal lobe and cortical midline regional dysfunction (reduced perfusion and activation), and with reduced parietotemporal metabolism. Reduced within and between network connectivity is observed in the default mode network regions of AD patients with anosognosia compared to AD patients without anosognosia and controls. During initial stages of cognitive decline in anosognosia, reduced indirect neural activity (i.e. perfusion, metabolism, and activation) is associated with the cortical midline regions, followed by the parietotemporal structures in later stages and culminating in frontotemporal dysfunction. Although the current evidence suggests differences in activation between AD or MCI patients with anosognosia and healthy controls, more evidence is needed exploring the differences between MCI and AD patients with and without anosognosia using resting state and task related paradigms.

**Electronic supplementary material:**

The online version of this article (10.1007/s11065-019-09410-x) contains supplementary material, which is available to authorized users.

## Introduction

The conceptualization of “unawareness dysfunction” by Gabriel Anton and Arnold Pick in 1882 focused on the awareness of illness in the mentally sick, thereby marking the start of the contemporary era of the neuropsychological study of anosognosia (Marková & Berrios, 2014). Joseph Babinski introduced the term anosognosia (from the Greek, α = without, νόσζ = disease, γνώσιζ = knowledge) in 1914 in Revue Neurologique. In his work, Babinski already offers insightful speculation that anosognosia may be specific to right hemispheric lesions and noted that, after some time, both described patients progressed to dementia (Langer & Levine, 2014). Recent research tends to define anosognosia by subtype or etiology: hemiplegic, cortical blindness, Anton’s syndrome, visual field defect, traumatic brain injury, multiple sclerosis, Parkinson’s disease, Alzheimer’s disease (AD), mild cognitive impairment (MCI), frontotemporal dementia and Huntington’s disease (Nurmi Laihosalo & Jehkonen, 2014; Prigatano, 2014). Patients with alterations in their self-awareness must show evidence of underlying brain pathology to be classified as having anosognosia or impaired self-awareness (Prigatano, 2014).

The cognitive decline continuum in Alzheimer’s disease can be divided into three stages, a preclinical, a prodromal and a clinical (Sperling et al., 2011). Mild cognitive impairment (MCI) is the transitional cognitive state between normal aging and mild dementia (Petersen et al., 2001). Of particular interest is amnestic mild cognitive impairment (aMCI) due to its emphasis on memory loss. While an important percentage of MCI patients remain stable for years or even revert to normal, patients with MCI, particularly aMCI, have a higher risk to progress to AD (Albert et al., 2011). In comparison to anosognosia due to focal lesions, anosognosia of memory deficits in dementia is less specific, in part, because patients underestimate their deficits in multiple domains (Wilson, Sytsma, Barnes, & Boyle, 2016). The incidence and prevalence of anosognosia of memory deficits has a large variability across dementia populations. The lack of a consensus in the diagnosis of anosognosia for memory deficits, as reflected by a large number of anosognosia screening instruments, contributes to the lack of specificity in the diagnosis and to the wide range in the prevalence of anosognosia in dementia. Factors that influence prevalence estimation are patient selection, which may be biased, assessment heterogeneity and lack of consensus on a severity scale (Wilson et al., 2016). Anosognosia for activities of daily living (ADL) deficits can be present from an early stage of AD and has a reported frequency between 20 and 80% (Starkstein, 2014). Patients with mild or moderate AD have a reported incidence between 21.0 and 38.3% and a prevalence between 24.2 and 71.0% for anosognosia (Starkstein, Brockman, Bruce, & Petracca, 2010; Castrillo-Sanz et al., 2016; Turró-Garriga et al., 2016). Cross-cultural assessment of the differences in unawareness of memory deficits in a large community-based study reports regional differences in the frequency of anosognosia, from 81.2% in India to 72.0% in Latin America and 63.5% in China (Mograbi et al., 2012). Recently, in an analysis of ADNI data, anosognosia has been identified as an independent predictor of conversion from MCI to AD (Gerretsen et al., 2017). Clinical data associate anosognosia to diverse dementias, and vice versa, clinical pathological studies suggest that dementia-related pathologies account for most cases of late-life anosognosia (Wilson et al., 2016).

While anosognosia is common in both AD and MCI patients, it is also associated with cognitive dysfunction and apathy in AD (Mak, Chin, Ng, Yeo, & Hameed, 2015; Spalletta, Girardi, Caltagirone, & Orfei, 2012). Furthermore, MCI patients underestimate their memory deficits (Vannini et al., 2017). Anosognosia of memory deficits is a clinically heterogeneous entity and can have a neuropsychological presentation that overlaps with apathy and depressive symptoms. However, to further understand the underpinnings of the differences between these neuropsychological symptoms, studies comparing amnestic or multiple domain MCI with and without anosognosia are needed. Anosognosia can be a byproduct of low-level perceptual deficits when it is associated with higher level deficits such as memory or intellectual impairments (Davies, Davies, & Coltheart, 2005; Turnbull, Fotopoulou, & Solms, 2014; Vuilleumier, 2004). There is no official method to diagnose anosognosia in AD, yet neuropsychiatric assessment by an experienced clinician complemented with additional information provided by an informant is considered the gold standard (Starkstein, 2014). Classification of anosognosia in a review of 64 studies in 2014 was assessed with 41 different methods, which reflects the lack of conceptual clarity and methodological consistency (Nurmi & Jehkonen, 2014). A gradual increase in the number of assessment batteries for anosognosia is reflected in the number of new measures that have become available in the last four decades, six new methods from 1978 to 1989 and 21 new methods from 2002 to 2013 (Nurmi Laihosalo & Jehkonen, 2014). Experimental assessment of anosognosia can be either direct or indirect, tailored to the subtype that the investigator wishes to evaluate. Because of the variability in diagnostic approaches, generalizations of results involving patients with anosognosia should be made carefully, as possible effects of patient selection, assessment methods, subtypes assessed and assessment time can impact the prevalence (Nurmi Laihosalo & Jehkonen, 2014; Orfei et al., 2010). Depression and anosognosia of memory deficits have been previously associated in patients with cognitive impairment. Patients with MCI without depressive symptoms can evaluate their memory impairment more accurately than those with depressive symptoms and patients with AD (Oba et al., 2018). Furthermore, patients who are aware of their memory loss are more likely to be depressed than those who suffer from anosognosia of memory deficits (Clare et al., 2012; Harwood, Sultzer, & Wheatley, 2000; Reed, Jagust, & Coulter, 1993). When compared to AD patients, patients with depression overestimate their memory abilities (Dalla Barba, Parlato, Iavarone, & Boller, 1995). However, knowledge of awareness of illness in mood disorders is limited and hence the assessment of directionality or reciprocal association between anosognosia of memory deficits and depression is currently unclear (Orfei, Robinson, Bria, Caltagirone, & Spalletta, 2008). Another factor that complicates interpretation of such association is the fact that depressive symptoms have been linked to other forms of cognitive decline (e.g. vascular dementia and frontotemporal dementia; De Carolis et al., 2015), while other studies have failed to associate anosognosia and depression (Mak et al., 2015; Spalletta et al., 2012). For additional factors that influence the level of awareness in MCI, we refer the reader to the work by Piras, Piras, Orfei, Caltagirone, and Spalletta (2016) and to the work of Orfei et al. (2008) for a discussion of awareness of illness in neuropsychiatric disorders.

Due to their high spatial resolution and low invasiveness, several functional neuroimaging techniques have been used to assess the neural correlates of anosognosia. Single photon emission computed tomography (SPECT) permits the measurement of regional cerebral blood flow, a measure of brain perfusion. Measurement of brain metabolism is possible with positron emission tomography (PET), while brain activation and connectivity can be determined with functional magnetic resonance imaging (fMRI). Impaired self-awareness and functional neuroimaging changes in cortical midline structures have previously been associated with neurodegenerative diseases in general, with dementia, and more specifically with AD. In neurodegenerative diseases (i.e. AD, frontotemporal dementia, Parkinson’s disease, Huntington’s disease, MCI and amyotrophic lateral sclerosis), impaired self-awareness has been linked to structural and functional neuroimaging abnormalities in the hippocampus, amygdala and temporopolar, entorhinal, perirhinal and posterior parahippocampal cortices (Chavoix & Insausti, 2017). In dementia patients, the neuroanatomical correspondents of unawareness are the frontal, medial parietal and lateral parietotemporal regions. These regions have been associated with the cognitive processing of self- and other-related information and are part of the default mode network (Zamboni & Wilcock, 2011). Alterations in cortical midline structures and default mode network intrinsic brain activity among AD patients have provided insight into the processing of self-related information (Weiler, Northoff, Damasceno, & Balthazar, 2016). Neuropathological, structural and functional changes in the medial temporal lobe have been found in patients with different neurodegenerative diseases who overestimate their performance in cognitive, socioemotional, or daily life activities (Chavoix & Insausti, 2017).

Previous neuroimaging reviews exploring the neural correlates of impaired self-awareness (Chavoix & Insausti, 2017) and brain correlates of unawareness of cognitive and behavioral symptoms (Zamboni & Wilcock, 2011) have attempted to associate deterioration of self-awareness to brain regions that show a diverse range of changes (functional, structural, and neuropathological) compared to persons without impaired self-awareness. In these reviews, the results from different neuroimaging techniques were combined, thereby strengthening the association between brain regions and impaired awareness by providing multiple perspectives to the same phenomenon. However, due to the intrinsic differences between neuroimaging techniques and interpretation of their results, analysis of the results based on technique rather than on changes in brain regions alone may provide a further understanding of anosognosia. Anosognosia in MCI and AD can be investigated in two different ways with functional neuroimaging. First by investigating self-referential tasks and secondly by studying connectivity. While task-related functional neuroimaging provides insight into functional segregation and localization of function, connectivity studies permit the study of neural processes in terms of functional integration.

Changes in the default mode network are detectable before dementia symptoms arise and functional connectivity is a promising biomarker for longitudinal studies in AD (Dennis & Thompson, 2014). Within and between network measures of brain connectivity obtained from fMRI allow for between-group comparisons. Network measures facilitate understanding of changes in the intranetwork and internetwork functional connectivity throughout the cognitive decline continuum (Zhu et al., 2016). Functional connectivity is defined as statistical dependencies among remote neurophysiological events (Friston, 2011). Functional MRI studies allow for the assessment of functional connectivity patterns associated with the generation and modulation of neural networks associated with decreased self-awareness in MCI and AD (Friston, 2011). While aging affects functional brain interactions, AD additionally specifically affects coherence between posterior default mode network and precuneus (Klaassens et al., 2017). Longitudinal functional connectivity data in AD patients compared to healthy subjects suggest disease-specific affected regions, namely, the frontoparietal network and precuneus (Hafkemeijer et al., 2017). The whole network analysis in the latter study revealed decreased mean connectivity in the frontoparietal network, while the network to region analyses reported a decrease over time in functional connectivity between the precuneus and the right frontoparietal network (Hafkemeijer et al., 2017). It has been suggested that the topological architecture of the functional connectome in amnestic MCI patients is disrupted and that its integrity is correlated to memory performance (Wang et al., 2013). Reduced regional resting state activity in amnestic MCI patients compared to healthy subjects has been found in the posterior cingulate cortex, right angular gyrus, right parahippocampal gyrus, left fusiform gyrus, left supramarginal gyrus and bilateral middle temporal gyri (Lau, Leung, Lee, & Law, 2016).

The aim of this review is to identify brain perfusion patterns, activation regions, and network connectivity characteristics that distinguish AD and MCI patients with anosognosia from healthy controls, as well as AD and MCI patients without anosognosia. To address this task, a systematic review was most appropriate. We contend that AD and MCI patients with anosognosia will have different brain perfusion patterns, activation patterns, and network connectivity compared to AD and MCI patients without anosognosia and healthy controls. These patterns will be characterized by topological changes affecting the posterior cingulate cortex, precuneus and angular gyrus (i.e. posterior default mode network) in early stages and the frontotemporal (i.e. anterior cingulate and medial prefrontal cortices) and parietotemporal regions (i.e. mediotemporal lobe and inferior parietal lobule), following a posterior to ventral, and anterior to dorsal gradient, in later stages. We expect that functional connectivity studies will provide further understanding, as network connectivity can serve as a potential biomarker for MCI (Franzmeier et al., 2017; Wang, Li, et al., 2013; Wang et al., 2013). A secondary aim of this review was to identify regional brain activation differences between self-appraisal task execution and resting state in AD and MCI patients with anosognosia and to provide a suitable conceptual model to explain these activation patterns. Activation differences in self-appraisal task execution and functional connectivity patterns observed in resting state fMRI have the potential to provide understanding of the self-referential processing of anosognosia in MCI and AD.

## Methods

### Study Selection

A systematic review of the literature was performed on PubMed, EMBASE, and PsycINFO databases in March 2018. Since the aim of the review was to identify the neural correlates of anosognosia, only indirect measures of neural activity were included in this review as the spatial resolution of these techniques is superior to that of direct techniques (e.g. electroencephalography and magnetoencephalography) which provide a better temporal resolution but a limited spatial resolution. The identification phase included no limit (i.e. any year, language, and publication status), which was followed by application of neuroimaging search terms. Identical no limits search strategies with a priori variables were realized on each database and reported according the Preferred Reporting Items for Systematic Reviews and Meta-Analyses statement (Moher et al., 2009) for the search terms anosognosia, self-appraisal, insight, awareness, and consciousness combined with each of the following terms: dementia, Alzheimer, and mild cognitive impairment. This broad no-limit search was initially performed using the search terms commonly associated with anosognosia or impaired awareness of memory loss as there is a heterogeneous reporting system for this clinical entity.

Following the initial search, neuroimaging search terms were applied to the results using the search terms: MRI, PET, SPECT, connectivity, activation, perfusion and metabolism. These search terms were selected to assure consistency with the aim of this review, which was to identify the neural correlates of anosognosia and self-awareness of memory loss through (indirect) neuroimaging techniques. The search terms connectivity, activation, perfusion, and metabolism were chosen to specifically target result characteristics of neuroimaging connectivity and metabolism studies. Specific sequences (e.g. Blood oxygen level dependent, diffusion tensor imaging, spectroscopy), modalities (e.g. fMRI both resting state and task related), and radioligands (e.g. 18F fluorodeoxyglucose, Pittsburgh compound B, Florbetapir, 123I iodoamphetamine, Technetium 99 m) were not chosen as search terms since one of the goals of this review was to include as many neuroimaging, connectivity and brain metabolism studies performed in human subjects as possible. A list of search terms and their combinations used in the search strategy can be found in the appendix (Supplemental Tables 6–8). Retrieved abstracts were screened by two of the authors to eliminate duplicate articles and articles not reporting neuroimaging data.

The resulting articles were selected for eligibility in a two-step process. First, by applying diagnosis and article type inclusion and exclusion criteria, done by two of the authors, followed by application of the neuroimaging inclusion and exclusion criteria, done by two of the authors. The inclusion criteria were: 1) articles published in English in a peer-reviewed journal, 2) human subjects diagnosed with AD according to the National Institute on Aging–Alzheimer’s Association (McKhann et al., 2011) or a previous version of these criteria (McKhann et al., 1984) or Diagnostic and Statistical Manual of Mental Disorders (all criteria), 3) MCI subjects diagnosed with the Petersen criteria (Petersen et al., 2001; Winblad et al., 2004) or National Institute on Aging–Alzheimer’s Association criteria update (Albert et al., 2011), 4) a validated screening method for anosognosia or task to assess self-appraisal was employed, 5) the results and discussion incorporated neuroimaging data and analysis, 6) the discussion associated neuroimaging results to anosognosia. The exclusion criteria were: 1) a review article, 2) inclusion of subjects with other neurodegenerative disorders (not including MCI or AD), 3) neuroimaging was only used as classification or screening instrument, 4) only structural imaging analysis was performed, 5) inclusion of subjects with language or comprehension impairment, and 6) inclusion of patients with known genetic risk for early-onset AD. The inter-rater agreement (i.e. Cohen’s kappa) was 0.902. Articles that required interpretation of their methods or had uncertain information (e.g. regarding self-awareness measurement technique) were reviewed by two of the authors (J.D.M and P.P.D.D.) and a consensus about their inclusion was reached. For the data collection process, all articles were available and downloaded from the University of Groningen Central Library databases and data extraction was undertaken by one of the authors and verified by the three authors.

### Data Assessment and Analysis

After eligibility assessment, a table extracting data from each article was created to evaluate the selected references. The articles were evaluated on the sociodemographic and clinical data, anosognosia assessment, neuroimaging analysis, and findings. Among the sociodemographic data evaluated were the type of population included (e.g. mild, moderate or severe stage of AD, MCI, amnestic MCI, healthy matched controls, young controls, AD with anosognosia, MCI with anosognosia), population size, age, male to female ratio, and education in years. The clinical data examined were the diagnostic criteria implemented for study inclusion, the cognition screening instrument used and operational definition parameters used to classify dementia, Mini mental state examination mean score, other neuropsychological assessments performed and whether the patients were taking any psychotropic medication (e.g. acetylcholinesterase inhibitors, selective serotonin reuptake inhibitors, antipsychotics, and benzodiazepines). Regarding anosognosia assessment, the measurement technique or questionnaire was noted, the method of awareness assessment was classified into discrepancy score between patient and informant or self-accuracy discrepancy score or expert classification only. All articles were classified based on the neuroimaging technique used (e.g. fMRI, PET or SPECT). Furthermore, the articles were classified based on the type of functional neuroimaging results reported (e.g. connectivity, activation or metabolism and perfusion).

After functional neuroimaging classification, a division between connectivity studies and metabolism studies (i.e. SPECT, PET, and activation only fMRI) was performed for further analysis. The functional neuroimaging results of each study included in the review were evaluated based on three characteristics: 1) the diagnosis of the population included, 2) the assessment method for awareness of memory deficit, 3) interpretation of the functional neuroimaging results. The Cochrane Collaboration recommends assessing the methodological quality of studies evaluating diagnostic tests using the following individual quality items: patient spectrum, reference standard, disease progression, partial verification, differential verification, test and diagnostic review, clinical review, uninterpretable results, and withdrawals (Reitsma et al., 2009). An open assessment of the risk of bias was performed based on the Cochrane Review Handbook for Diagnostic Test Accuracy (The Cochrane Collaboration, London). The modified Quality Assessment of Diagnostic Accuracy Studies checklist part of the Cochrane RevMan 5.3 software (The Nordic Cochrane Center, Copenhagen 2019) was used to assess the internal validity of each study included in the review. Decisions about the risk of bias items that required judgment or interpretation were discussed and a consensus was reached between the authors. To assess external validity of the articles included in this review, risk of bias tables were generated (Supplemental Table 1 and Supplemental Figs. 1 and 2) to examine the tendencies and future direction of research. For discussion purposes, we review the results according to study design. Two different study designs are included in this review, case-control studies (i.e. dichotomization into two groups, anosognosia or without anosognosia) and studies that correlate awareness of memory deficit with clinical status (i.e. correlation of awareness to brain perfusion, metabolism, activation or connectivity). Case-control studies comparing MCI or AD patients with anosognosia (the cases) to MCI or AD patients without anosognosia (the controls) allow for a dichotomized comparison of awareness of memory deficits. For these studies, we will refer to the impairment of memory awareness as anosognosia. This approach has the advantage of detecting specific regional differences in neural correlates of anosognosia, yet with limited capacity to assess awareness of memory deficits continuously. Similarly, we will refer to the impairment of memory awareness as unawareness of memory deficit to those studies that measure awareness as a continuous variable and studies that correlate awareness to indirect measures of neural activity (i.e. neuroimaging outcomes). In contrast to anosognosia studies that focus on unawareness status, unawareness of memory deficit studies provide a continuous perspective on anosognosia, nonetheless, with a limited proficiency to differentiate cases from controls.

## Results

**Fig. 1:**
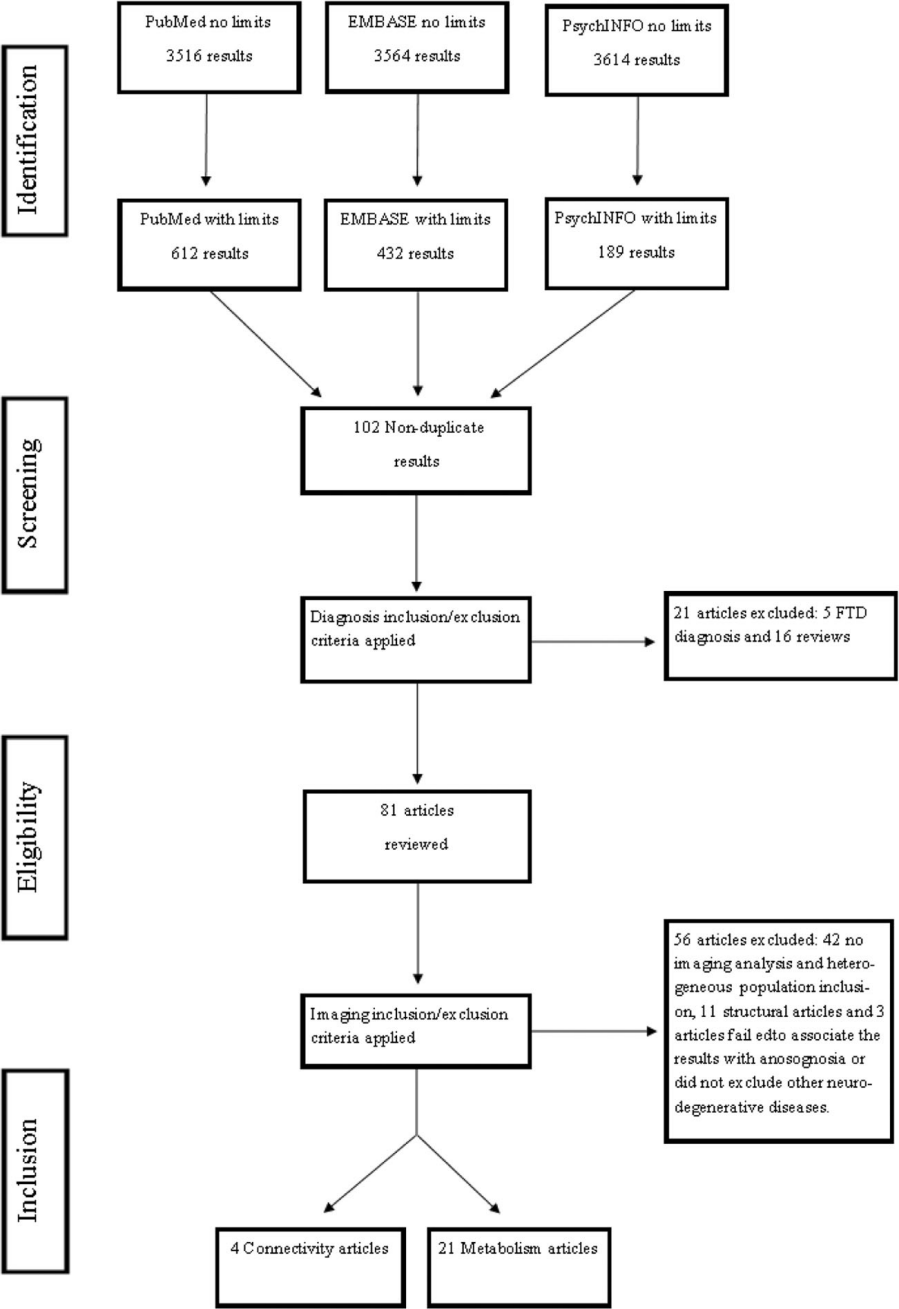
Preferred Reporting Items for Systematic Reviews and Meta-Analyses patient selection flowchart. FTD: frontotemporal demetia

The without-limit review of the literature yielded 3516 results from PubMed, 3564 results from EMBASE and 3614 results from PsychINFO. Figure 1 shows the Preferred Reporting Items for Systematic Reviews and Meta-Analyses flowchart of the selection process. After the neuroimaging search terms were applied, database results narrowed to 612 results for PubMed, 432 results for EMBASE and 189 results for PsychINFO. The titles and abstracts of the with-limits results were reviewed, yielding 102 articles not duplicated. These articles were screened for eligibility by applying diagnosis and article type inclusion and exclusion criteria. Twenty-one articles were excluded, as five studied patients with frontotemporal dementia and 16 were review articles. In the second step of the eligibility process, the remaining 81 articles were further assessed and neuroimaging inclusion and exclusion criteria were applied. Fifty-six articles were excluded, with 42 articles not having a validated screening method for anosognosia or not having neuroimaging analysis and another 11 articles focusing on structural neuroimaging techniques. The final three articles were excluded during the data extraction process after a consensus was reached that two (Genon et al., 2014; Gaubert et al., 2017) failed to associate neuroimaging results with anosognosia in their discussion, while one study included patients with clinical and imaging findings suggesting other neurodegenerative or vascular pathologies into the imaging analysis (Ott, Noto, & Fogel, 1996). Twenty-five articles met all inclusion parameters and were further evaluated in this systematic review, specifically, four brain connectivity and 21 brain perfusion, metabolism, and activation articles. The references of the included articles were examined in a search for additional literature, yielding no additional studies. A meta-analysis, however, could not be performed, as we found that the studies being evaluated lacked sufficient similarity regarding the population, anosognosia assessment method, and neuroimaging outcome measures to justify the statistical combination of the results.

### Study Characteristics

#### Brain Perfusion

Brain perfusion was investigated using SPECT in ten articles. A summary of SPECT study characteristics may be found in Table 1. Five used N-isopropyl-p-[123I]-iodoamphetamine (Derouesné et al., 1999; Hanyu et al., 2008; Reed et al., 1993; Shibata et al., 2008; Tagai et al., 2018), four used Technetium-99 m-hexamethylpropyleneamine oxime (Sedaghat et al., 2010; Starkstein et al., 1995, 1996; Vogel et al., 2005) and one used Technetium-99 m-ethyl-cysteinate dimer (Mimura & Yano, 2006) as radioligands. These studies included heterogeneous population samples. One article compared AD patients to healthy controls (Mimura & Yano, 2006) and four articles contrasted AD patients with and without anosognosia or aware versus unaware of memory deficits patients (Hanyu et al., 2008; Sedaghat et al., 2010; Starkstein et al., 1995; Tagai et al., 2018). Three articles were observational studies that correlated awareness performance and regional cerebral blood flow in the AD population (Derouesné et al., 1999; Reed et al., 1993; Shibata et al., 2008), one carried out this same correlation comparing AD and ischemic vascular dementia patients (Starkstein et al., 1996) and another compared mild AD versus amnestic MCI (Vogel et al., 2005). The cognition screening instruments used were the Mini mental state examination in all SPECT studies while five additionally used the Clinical Dementia Rating for patient severity classification (Hanyu et al., 2008; Mimura & Yano, 2006; Sedaghat et al., 2010; Vogel et al., 2005; Tagai et al., 2018).

**Table 1:** Characteristics of brain perfusion studies

Reference	Population	Study population		MMSE		Anosognosia measurement instrument	Neuroimage technique	Findings
		Interest	HC	Interest	HC			
Reed et al., 1993	AD	20	NA	19.5 (4.5)	NA	Anosognosia clinical rating scale	SPECT (123I-IMP)	Hypoperfusion of right dorsolateral frontal lobe.
Starkstein et al., 1995	AD	12 AD anosognosia	NA	18.5 (4.7)	NA	AQ-D	SPECT (99mTc - HMPAO)	Hypoperfusion of right frontal lobe (frontal inferior and frontal superior).
		12 AD without anosognosia		19.1 (5.6)				
Starkstein et al., 1996	Ischemic vascular dementia vs AD	20 AD	NA	19.9 (6.3)	NA	AQ-D	SPECT (99mTc - HMPAO)	IVD patients showed hypoperfusion in frontal regions and basal ganglia compared with AD patients.
		10 IVD		19.0 (7.1)				
Derouesné et al., 1999	Mild AD	78	NA	22.5 (3.2)	NA	Cognitive Difficulties Scale and Anosognosia clinical rating scale	SPECT (123I-IMP)	Hypoperfusion of frontal regions correlated to decreased awareness.
Vogel, Hasselbalch, Gade, Ziebell, & Waldemar, 2005	Mild AD vs aMCI	30 AD	NA	24 (2.5)	NA	Memory Questionnaire	SPECT (99mTc - HMPAO)	Hypoperfusion of right inferior frontal gyrus in AD/aMCI patients with anosognosia.
		25 aMCI		26 (2.06)				
Mimura et al., 2006	Mild and moderate AD	24	20	22.3 (3.8)	28.8 (1.0) HC1; NA for HC2	Awareness of memory experimental paradigm	SPECT (99mTc-ECD)	Positive correlations between awareness performance and rCBF in the medial frontal lobe, right precuneus and inferior frontal gyrus found in aware controls when compared to AD group.
Hanyu et al., 2008	AD aware vs unaware	19 AD aware	NA	25.5 (1.1)	NA	Every day Memory Checklist	SPECT (123I-IMP)	Hypoperfusion of lateral and medial frontal lobes, anterior cingulate and cingulate gyri of both hemispheres and the inferior parietal region of the left hemisphere.
		19 AD unaware		25.8 (1.3)				
Shibata, Narumoto, Kitabayashi, Ushijima, & Fukui, 2008	AD	29	NA	21.2 (2.9)	NA	Questionnaire adapted from Squire and Zouzounis	SPECT (123I-IMP)	Hypoperfusion of bilateral orbitofrontal cortex (simple regression between rCBF and anosognosia scores).
Sedaghat et al., 20101.	AD with vs without anosognosia	22 AD anosognosia	NA	18 (4)	NA	Non-structured interview	SPECT (99mTc - HMPAO)	Hypoperfusion of right prefrontal, right inferior parietal and bilateral medial temporal cortex.
		20 AD without anosognosia		21 (4)				
Tagai et al., 2018	AD with vs without anosognosia	11 AD anosognosia	NA	20.4 (4.6)	NA	AQ-D	SPECT (123I-IMP)	Hypoperfusion of right prefrontal cortex and Hyperperfusion of left temporo-parietal junction
		20 AD and 6 MCI without anosognosia		20.4 (4.6)				

*123I-IMP* N-isopropyl-p-[123I]-iodoamphetamine, *99mTc-ECD* Technetium-99 m-ethyl-cysteinate dimer, *99mTc-HMPAO* Technetium-99 m-hexamethylpropyleneamine oxime, *AD* Alzheimer’s disease, *aMCI* amnestic mild cognitive impairment, *AQ-D* Anosognosia Questionnaire Dementia, *HC* healthy controls, *IVD* ischemic vascular dementia, *MMSE* Mini-mental state examination, *NA* Not applicable or not available, *SPECT* single-photon emission computed tomography

The measurement instruments used to assess anosognosia were the Anosognosia Questionnaire for Dementia in three articles (Starkstein et al., 1995, 1996; Tagai et al., 2018), a customized anosognosia clinical rating scale (Reed et al., 1993), the Memory Questionnaire (Vogel et al., 2005), an awareness of memory experimental paradigm based on the Auditory Verbal Learning Test (Mimura & Yano, 2006), the Everyday Memory Checklist (Hanyu et al., 2008), a questionnaire adapted from Squire and Zouzounis’ (Shibata et al., 2008), multiple interviews (Sedaghat et al., 2010) and a combination of anosognosia clinical rating scale and the Cognitive Difficulties Scale discrepancy score (Derouesné et al., 1999). Anosognosia Questionnaire for Dementia is a 30-item scale measuring awareness of functional deficits and behavioral changes in which the responses from the patient are compared to those from the informant (Migliorelli et al., 1995). The Everyday Memory Checklist is a questionnaire consisting of 13 questions concerning areas of daily life, while the Squire and Zouzounis questionnaire includes 20 items concerned with aspects of memory function, both questionnaires are administered and scored in a similar manner as the Anosognosia Questionnaire for Dementia (Shibata et al., 2008; Wilson, Cockburn, Baddeley, & Hiorns, 1989). The customized anosognosia clinical rating scale used by Reed et al. (1993), which was later adapted by Vogel and colleagues (Vogel et al., 2005) as the Memory Questionnaire, is a categorical four-point scale where a clinical neuropsychologist or clinical expert rates the overall impression of the level of awareness into: ‘Full awareness’, ‘shallow awareness’, ‘no awareness’, and ‘denies impairment’. The Auditory Verbal Learning Test was used to compare the prediction and postdiction performance on the 15-item list of words recognition test as an awareness of memory experimental paradigm. The Cognitive Difficulties Scale is a 37-item self-rated questionnaire which assesses the experience with everyday life activities, the index used by Derouesné and colleagues (Derouesné et al., 1999) assesses unawareness of cognitive deficits like Anosognosia Questionnaire for Dementia.

#### Brain Metabolism

Seven studies correlated 18F fluorodeoxyglucose brain metabolism to the level of awareness of memory deficit. A summary of PET study characteristics may be found in Table 2. Two studies included exclusively mild to moderate AD patients (Harwood et al., 2005; Salmon et al., 2006), one study included mild to severe AD patients (Sultzer et al., 2014), another compared early AD patients to healthy controls (Jedidi et al., 2014), one study compared both early AD and amnestic MCI to healthy controls (Gerretsen et al., 2017), and two studies compared aware versus unaware of memory deficits patients with amnestic MCI to healthy controls (Nobili et al., 2010; Therriault et al., 2018). The cognition screening instruments used were the Mini mental state examination (Harwood et al., 2005), the Clinical Dementia Rating (Nobili et al., 2010; Salmon et al., 2006), the Mattis dementia rating scale (Jedidi et al., 2014; Sultzer et al., 2014), while one study used the combination of the Mini mental state examination and Clinical Dementia Rating (Therriault et al., 2018), and another a combination of the Mini mental state examination, Montreal Cognitive Assessment and Clinical Dementia Rating (Gerretsen et al., 2017). Anosognosia was assessed using the inaccurate insight item of the Neurobehavioral Rating Scale (Harwood et al., 2005; Sultzer et al., 2014), an experimental questionnaire designed for the Network for Efficiency and Standardization of Dementia Diagnosis (Salmon et al., 2006), the Memory Complaint Questionnaire (Nobili et al., 2010), the Klein and colleagues’ personality traits questionnaire (Jedidi et al., 2014), and the Everyday Cognition Scale (Gerretsen et al., 2017; Therriault et al., 2018). The inaccurate insight item of the Neurobehavioral Rating Scale is based on 12 items where the patient’s ideas and plans are contrasted with objective information obtained by the examiner from the clinical interview, cognitive testing and informant report (Harwood et al., 2005; Sultzer et al., 2014).

**Table 2:** Characteristics of brain metabolism studies

Reference	Population	Study population		MMSE		Anosognosia measurement instrument	Neuroimage technique	Findings
		Interest	HC	Interest	HC			
Harwood et al., 2005	Mild to moderate AD	41	NA	19.3 (6.7)	NA	Inaccurate Insight item of the Neurobehavioral rating scale	FDG-PET	Hypometabolism in the right lateral frontal cortex associated with inaccurate insight.
Salmon et al., 2006	Mild to moderate AD	209	NA	21.0 (4.5)	NA	Experimental questionnaire for the NEST-DD	FDG-PET	Hypometabolism in the orbital prefrontal cortex and medial temporal structures.
Nobili et al., 2010	aMCI aware vs unaware	17 aMCI unaware	29	28.1 (1.6)	29.2 (1.1)	Memory Complaint Questionnaire	FDG-PET	Correlation between hypometabolism and awareness in bilateral posterior cingulate cortex and inferior parietal lobule, middle cingulate cortex, precuneus and angular gyrus in left hemisphere among aMCI group. Hypometabolism in precuneus, inferior parietal lobe and superior occipital gyrus in the left hemisphere and inferior parietal lobe, angular gyrus and middle temporal gyrus in the right hemisphere in aMCI/unaware compared to controls. Hypometabolism in bilateral temporal lobe in aMCI/aware compared to controls. Hypometabolism in inferior parietal lobule, angular gyrus, and superior temporal gyrus in the left hemisphere in aMCI/unaware versus aMCI/aware.
		25 aMCI aware		28.0 (1.7)				
Jedidi et al., 2014	“early” AD	37	25	NA	NA	Klein and colleagues’ personality traits questionnaire	FDG-PET	Hypoactivation of the dorsomedial prefrontal cortex is negatively correlated with anosognosia for current personality traits.
Sultzer et al., 2014	AD	80	NA	19.3 (5.1)	NA	Inaccurate insight item of Neurobehavioral Rating Scale	FDG-PET	Hypometabolism in bilateral medial frontal cortex correlated to poorer insight according to the Neurobehavioral Rating Scale inaccurate insight item.
Garretsen et al., 2017	AD and MCI	191 AD	372	22.4 (3.0)	29 (1.3)	Everyday Cognition scale	FDG-PET	Hypometabolism in posterior cingulate cortex and right angular gyrus in AD.
		499 MCI		28.1 (1.7)				
Therriault et al., 2018	MCI	175 aMCI unaware	NA	NA	NA	Everyday Cognition scale	FDG-PET	Hypometabolism in the posterior cingulate cortex, left basal forebrain, bilateral temporal lobes, and right lateral temporal lobe associated withimpaired awareness in aMCI over 24 months.
		293 aMCI aware		NA				

*AD* Alzheimer’s disease, *aMCI* amnestic mild cognitive impairment, *FDG-PET* 18F-fluorodeoxyglucose positron emission tomography, *HC* healthy controls, *MMSE* Mini-mental state examination, *NA* Not applicable or not available, *NEST-DD* Network for Efficiency and Standardization of Dementia Diagnosis

The experimental questionnaire used by Salmon and colleagues (Salmon et al., 2006) studies multiple symptoms associated with dementia and uses a discrepancy score between the self-evaluation score from the AD patient and the informant total score. The Memory Complaint Questionnaire is a six questions self-evaluation questionnaire designed to evaluate memory decline associated with aging by comparing the present and past status of daily activities and global memory functions (Crook, Feher, & Larrabee, 1992). The Klein and colleagues’ personality traits questionnaire assesses the patient’s ability to judge their own personality in the present and obtains the difference in score with the informant’s responses about the patient’s personality traits (Jedidi et al., 2014). The Everyday Cognition Scale measures global as well as specific cognitive functions (e.g. episodic memory and planning) based on the perception of present cognitive abilities compared to those same abilities 10 years earlier.

#### Brain Activation

Brain activation was explored through task-related fMRI by four studies. The first study uses a self-appraisal task (Ries et al., 2007), while the second study uses a personality self-appraisal versus other-appraisal task (Ruby et al., 2009). The third study explores activation using an inhibition task (Amanzio et al., 2011), while the last study used a cognitive, behavioral and physical trait self-appraisal versus other-appraisal task (Zamboni et al., 2013). A summary of fMRI brain activation study characteristics may be found in Table 3. These studies include a vast population spectrum, ranging from a general disease perspective comparing AD patients to both healthy old and young controls (Ruby et al., 2009) to a specific population selection by comparing AD patients with and without anosognosia (Amanzio et al., 2011). MCI patients were also studied by Ries and colleagues (Ries et al., 2007) who compared MCI to healthy controls and Zamboni and colleagues (Zamboni et al., 2013) who compared AD and MCI patients to healthy controls. The cognition screening instruments used were Mini mental state examination (Amanzio et al., 2011; Ries et al., 2007; Zamboni et al., 2013), Clinical Dementia Rating (Ruby et al., 2009), and Montreal Cognitive Assessment (Zamboni et al., 2013). Anosognosia Questionnaire for Dementia was used to assess anosognosia by two articles (Amanzio et al., 2011; Zamboni et al., 2013), the Klein and colleagues’ personality traits questionnaire by one article (Ruby et al., 2009), and the Informant Questionnaire on Cognitive Decline in the Elderly by another (Ries et al., 2007). The Informant Questionnaire on Cognitive Decline in the Elderly is a 16-item instrument that rates the patient’s cognitive changes in the last 10 years and assesses awareness is a discrepancy score between the patient and the informant, like the Anosognosia Questionnaire for Dementia (Jorm, 2004).

**Table 3:** Characteristics of brain activation studies

Reference	Population	Study population		MMSE		Anosognosia measurement instrument	Neuro image technique	Findings
		Interest	HC	Interest	HC			
Ries et al., 2007	MCI	16	16	27.4 (2.2)	29.7 (0.4)	IQCODE	Self-appraisal task fMRI	Attenuated activation of mPFC and PCC.
Ruby et al., 2009	AD vs healthy controls; young controls vs elderly controls	14	17	NA	NA	Personality awareness score (Klein and colleagues’ personality traits questionnaire)	Personality self-appraisal versus other appraisal task fMRI	Intraparietal sulcus activated during self-processing; a region involved in familiarity-based retrieval of information. Impaired third-person perspective taking associated with increased activation of prefrontal cortex.
			17 young					
Amanzio et al., 2011	AD with vs without anosognosia	14 AD with anosognosia	NA	22.2 (2.0)	NA	AQ-D	Inhibition task (go-no go) fMRI	Reduced activation of the right post-central gyrus (BA 2), right parietotemporal-occipital junction (BA 39) and the left temporal gyrus (BA 21 and BA 38), striatum and cerebellum. Activation of posteriormedial parietal areas
		15 AD without anosognosia		22.5 (2.2)				
Zamboni et al., 20131.	AD vs MCI	17 AD	17	22.2 (3.0)	29.9 (0.7)	AQ-D	Cognitive, behavioral and physical self-appraisal versus other appraisal task fMRI	AD patients with decreased functional activation of medial prefrontal and anterior temporal cortices; specific for self but not for other appraisal task. MCI patients’ activation like controls.
		17 MCI		26.8 (1.4)				

*AD* Alzheimer’s disease, *AQ-D* Anosognosia Questionnaire Dementia, *BA* Brodmann area, *fMRI* functional magnetic resonance imaging, *HC* healthy controls, *IQCODE* Informant Questionnaire on Cognitive Decline in the Elderly, *mPFC* medial prefrontal cortex, *MCI* mild cognitive impairment, *MMSE* Mini-mental state examination, *NA* Not applicable or not available, *PCC* posterior cingulate cortex

#### Brain Connectivity

Brain connectivity was investigated by four articles (Berlingeri et al., 2015; Perrotin et al., 2015; Ries et al., 2012; Vannini et al., 2017). A summary of brain connectivity study characteristics may be found in Table 4. The functional neuroimaging techniques used by the three connectivity studies were self-appraisal task fMRI (Ries et al., 2012) and resting state fMRI (Berlingeri et al., 2015; Perrotin et al., 2015; Vannini et al., 2017). All of the previous studies used region of interest connectivity analysis. The populations studied in these three articles ranged from mild AD patients (Perrotin et al., 2015), and amnestic MCI patients (Vannini et al., 2017), to a mixture of mild AD and MCI patients (Ries et al., 2012), and AD patients with and without anosognosia (Berlingeri et al., 2015). The cognition screening instruments used were the Clinical Dementia Rating (Ries et al., 2012), the Mini mental state examination (Berlingeri et al., 2015; Perrotin et al., 2015), and a combination of the Mini mental state examination and Clinical Dementia Rating (Vannini et al., 2017).

**Table 4:** Characteristics of brain connectivity studies

Reference	Population	Study population		MMSE		Anosognosia measurement instrument	Neuro image technique	Findings
		Interest	HC	Interest	HC			
Ries et al., 2012	AD/MCI	5 AD	12	25* [17–30]	30* [29–30]	Memory Awareness Rating Scale	Self-appraisal task fMRI	Attenuated functional connectivity between mPFC and proximal areas, bilateral dorsolateral prefrontal cortex, bilateral caudate and left posterior hippocampus.
		7 MCI		25* [17–30]				
Berlingeri et al., 2015	AD with vs without anosognosia	10 with anosognosia	15	24.5 (2.92)	28.87 (1.25)	AQ-D	rsfMRI	Reduced functional connectivity within DMN, within network comprised of the lateral temporal cortex, the hippocampus and the insula, and reduced connectivity between hippocampus and insular cortex.
		8 without anosognosia						
Perrotin et al., 2015	AD	23	30	21.52 (4.62) [12–29]	29.17 (0.83) [28–30]	Self-Rating Scale of Memory Function	rsfMRI/ FDG-PET	Hypometabolism in orbitofrontal (OFC) and posterior cingulate (PCC) cortices; reduced intrinsic connectivity between OFC and medial temporal lobe (MTL) and PCC and MTL.
Vannini et al., 2017	aMCI	31	251	27.1 (1.9)	28.9 (1.1)	Memory Functioning Questionnaire	rsfMRI/ FDG-PET	Hypometabolism in precuneus and hippocampus. Reduced functional connectivity between precuneus and bilateral inferior parietal lobes (IPL), left PCC, left OFC. Reduced functional connectivity between right hippocampus and left MTL and right fusiform gyrus.

*AD* Alzheimer’s disease, *AQ-D* Anosognosia Questionnaire Dementia, *DMN* default mode network, *FDG-PET* 18F-fluorodeoxyglucose positron emission tomography, *fMRI* functional magnetic resonance imaging, *HC* healthy controls, *MCI* mild cognitive impairment, *MMSE* Mini-mental state examination; *rs-fMRI* resting state fMRI

*Median and range

The measurement instruments used to assess anosognosia were the Anosognosia Questionnaire for Dementia (Berlingeri et al., 2015), the Memory Awareness Rating Scale (Ries et al., 2012), and a discrepancy score derived from the Self-Rating Scale of Memory Function (Perrotin et al., 2015) and the Memory Functioning Questionnaire (Vannini et al., 2017). The Memory Awareness Rating Scale is a psychometric test that assesses awareness of the patient’s ability to perform memory tasks during everyday activities by calculating a discrepancy score between the patient’s self-appraisal score and an informant’s parallel questionnaire score (Clare, Wilson, Carter, Roth, & Hodges, 2002). The discrepancy score is an anosognosia index that is calculated by the subtraction of standardized objective from subjective memory scores with the resulting outcome indicating the degree of anosognosia, represented by negative values (Dalla Barba et al., 1995).

### Functional Neural Correlates

#### Brain Perfusion

Observational anosognosia SPECT studies provide an understanding of how the awareness gradient in the general AD population relates to brain perfusion. However, case-control studies employ selective sampling comparing aware versus unaware of memory deficits patient groups, thus presenting accentuated between-group differences. Three observational studies correlated awareness performance and regional cerebral blood flow in the AD population. Altogether, anosognosia is associated with hypoperfusion in bilateral frontal regions (Derouesné et al., 1999), the right dorsolateral frontal lobe (Reed et al., 1993) and bilateral, superior, medial, inferior frontal and orbitofrontal cortex (Shibata et al., 2008). Another observational study, incorporating both mild AD and amnestic MCI patients, reported hypoperfusion of right inferior frontal gyrus in AD and amnestic MCI patients associated with impaired awareness of memory deficits (Vogel et al., 2005). Impaired recognition memory and recognition performance correlated negatively with regional cerebral blood flow in the medial frontal lobe, inferior frontal lobe, and right precuneus in AD patients when compared to healthy controls (Mimura & Yano, 2006). Four studies compare AD patients with and without anosognosia (Sedaghat et al., 2010; Starkstein et al., 1995; Tagai et al., 2018) or aware versus unaware of memory deficit patients (Hanyu et al., 2008). AD patients with anosognosia exhibit hypoperfusion in the bilateral medial temporal regions, right inferior parietal cortex and right parietotemporal cortex (Sedaghat et al., 2010), right frontal inferior and superior areas (Starkstein et al., 1995), and right prefrontal cortex (Tagai et al., 2018). Compared to AD patients without anosognosia, patients unaware of memory deficits show hypoperfusion in the inferior, medial and orbital frontal lobes and anterior cingulate gyri (Hanyu et al., 2008). The last SPECT study included in this review compares AD and ischemic vascular dementia patients, reporting that vascular dementia patients showed hypoperfusion in frontal regions and basal ganglia compared with AD patients (Starkstein et al., 1996).

- **Summary:** In MCI patients, anosognosia is associated with hypoperfusion in the bilateral lateral and medial frontal lobes, the bilateral anterior cingulate cortex and cingulate gyri, and the left inferior parietal region. Unawareness of memory deficits in MCI is correlated to lower perfusion in the right inferior frontal gyrus. In AD patients, hypoperfusion in the right frontal lobe, the right inferior parietal, bilateral medial temporal cortex, right prefrontal cortex and hyperperfusion of left temporo-parietal junction is observed when anosognosia of memory deficits is present. In regard to unawareness of memory deficits in mild to moderate AD, hypoperfusion is observed in the frontal regions bilaterally, the right dorsolateral frontal lobe, the right precuneus, and right inferior frontal gyrus.

#### Brain Metabolism

The neurobiological substrate of impaired insight in AD patients can also be studied through glucose brain metabolism. MCI patients are included in three studies (Gerretsen et al., 2017; Nobili et al., 2010; Therriault et al., 2018). One 18F fluorodeoxyglucose PET study included early AD patients (Jedidi et al., 2014), two studies included mild to moderate AD patients (Harwood et al., 2005; Salmon et al., 2006), and one study considered mild to severe AD patients (Sultzer et al., 2014). A significant association between inaccurate insight and hypometabolism in AD patients was found in the right lateral frontal lobe (Harwood et al., 2005). Salmon and colleagues (Salmon et al., 2006) associate impaired self-evaluation with lower metabolic activity in the orbital prefrontal cortex and medial temporal structures in mild to moderate AD patients. In moderate to severe dementia, patients’ lower cortical metabolic activity in the bilateral medial frontal cortex was associated with poorer insight according to the Neurobehavioral Rating Scale inaccurate insight item (Sultzer et al., 2014). When brain metabolism was compared between early AD patients and healthy controls, hypoactivation of the dorsomedial prefrontal cortex (Jedidi et al., 2014) and lower glucose metabolism in the posterior cingulate cortex (Gerretsen et al., 2017; Perrotin et al., 2015; Therriault et al., 2018), the precuneus and the medial orbitofrontal cortex (Perrotin et al., 2015) was found. Like early AD patients, when compared to healthy controls, amnestic MCI patients’ show reduced metabolism in the bilateral posterior cingulate cortex and inferior parietal lobule (Gerretsen et al., 2017; Nobili et al., 2010). Furthermore, the left hemisphere showed reduced metabolism in the middle cingulate cortex, precuneus, and angular gyrus (Gerretsen et al., 2017; Nobili et al., 2010; Vannini et al., 2017). Nobili and colleagues (Nobili et al., 2010) also reported hypometabolism in the temporal lobe bilaterally when comparing amnestic MCI aware patients and healthy controls. Although aware versus unaware of memory deficits amnestic MCI patients shared extended low metabolic regions, unaware amnestic MCI patients showed distinct hypometabolism in these typical AD regions especially in the precuneus, inferior parietal lobe and superior occipital gyrus in the left hemisphere and inferior parietal lobe, angular gyrus and middle temporal gyrus in the right hemisphere (Nobili et al., 2010). Lower metabolic activity is found in the inferior parietal lobule, angular gyrus, and superior temporal gyrus in the left hemisphere in unaware amnestic MCI patients when compared to aware amnestic MCI patients (Nobili et al., 2010). When compared to healthy controls, patients with MCI and anosognosia have hypometabolism in the right hippocampus and precuneus (Vannini et al., 2017). Hypometabolism in the left basal forebrain, bilateral temporal lobes, and right lateral temporal lobe is associated with impaired awareness in amnestic MCI patients who develop anosognosia over a period of 24 months (Therriault et al., 2018). Furthermore, poor awareness of cognitive deficit has been associated with subsequent conversion to AD (Spalletta et al., 2014).

- **Summary**: In MCI patients, anosognosia is associated with hypometabolism of the posterior cingulate cortex, precuneus, right hippocampus, bilateral temporal cortex, left inferior parietal lobule, the left angular gyrus, and the left superior temporal gyrus. Meanwhile, lower glucose metabolism in the left precuneus, left inferior parietal lobe and left superior occipital gyrus, right inferior parietal lobe, right angular gyrus, and right middle temporal gyrus are correlated to unawareness of memory deficits in MCI. In AD patients, anosognosia is associated with hypometabolism in posterior cingulate cortex and right angular gyrus. Unawareness of memory deficits in mild to moderate AD is correlated to hypometabolism of the bilateral medial prefrontal cortex, bilateral orbitofrontal cortex and posterior cingulate cortices, the right lateral frontal cortex, the right parahippocampal cortex, the right gyrus rectus, the right middle temporal cortex, left superior frontal sulcus, left dorsomedial prefrontal cortex.

#### Brain Activation

Brain activation evaluated with fMRI can be achieved through resting state and task-related functional neuroimaging. Execution of self-processing tasks, provides contrasting evidence to resting state fMRI data in dementia patients with anosognosia, as increased activation is observed in the former, rather than hypoactivation observed in the latter. During self-processing, mild AD patients compared to healthy controls have increased activation of the intraparietal sulcus, a region involved in retrieval of familiar information assessed through a self-personality awareness task (Ruby et al., 2009). Furthermore, this study also reports the association of impaired third-person perspective taking with increased activation of the prefrontal cortex (Ruby et al., 2009). MCI patients with reduced insight show attenuated medial prefrontal cortex and posterior cingulate cortex activity compared to controls during a self-appraisal task. However, fMRI activation and level of self-awareness are not correlated to the level of cognitive impairment (Ries et al., 2007). AD patients with anosognosia have reduced activation in the medial prefrontal cortex during a self-appraisal task compared to MCI patients and healthy controls. Concurrently, AD patients fail to activate the anterior temporal lobe during self-appraisal (Zamboni et al., 2013). In AD patients with anosognosia performing a binary classification task, reduced activation is reported in the cingulofrontal and parietotemporal regions compared with AD patients without anosognosia (Amanzio et al., 2011).

- **Summary**: In MCI, unawareness of memory deficits is correlated with lower activation in the bilateral medial prefrontal and posterior cingulate cortices. Meanwhile, anosognosia is associated with hypoactivation in the right postcentral gyrus, right parietotemporal and parietooccipital junction and the left temporal gyrus, striatum and cerebellum for case-control studies in mild to moderate AD patients. In mild to moderate AD, hypoactivation of the bilateral dorsomedial prefrontal cortex, bilateral medial prefrontal cortex, bilateral anterior temporal cortices and hyperactivation of intraparietal sulcus are correlated to unawareness of memory deficits.

#### Brain Connectivity

Compared to healthy controls, dementia patients with anosognosia have reduced within-network functional connectivity in the lateral middle temporal cortex network (i.e. superior frontal gyrus, precentral gyrus, supplementary motor area, insular cortex, postcentral gyrus, superior parietal gyrus, middle cingulum, paracentral lobule, precuneus, superior temporal pole, superior temporal gyrus, middle temporal gyrus, inferior temporal gyrus, amygdala, parahippocampal gyrus, anterior hippocampus, middle hippocampus, lingual gyrus, thalamus, putamen and cerebellum). While dementia patients without anosognosia have reduced within-network functional connectivity in the default mode network, compared to healthy controls (Berlingeri et al., 2015). Reduced functional connectivity between precuneus and bilateral inferior parietal lobes, left posterior cingulate cortex, and left orbitofrontal cortex was reported in patients with amnestic MCI and anosognosia when compared to healthy controls (Vannini et al., 2017). Reduced between-network functional connectivity between the right hippocampus and left mediotemporal lobe and right fusiform gyrus is also found in amnestic MCI patients with anosognosia when compared to healthy controls (Vannini et al., 2017). Reduced between-network functional connectivity is reported between the lateral middle temporal cortex network and the lateral temporal cortices, the medial temporal structures, the retrosplenial and midline structures, and the frontal and insular areas in dementia patients with anosognosia compared to AD patients without anosognosia and healthy controls. Additionally, anosognosia deficit severity was correlated to reduced functional connectivity between the lateral middle temporal cortex network and the middle hippocampal regions, and insular cortex (Berlingeri et al., 2015). Interestingly, no correlation between severity of anosognosia and within-network functional connectivity in the default mode network is reported (Berlingeri et al., 2015). Reduced connectivity between the medial temporal lobe and two regions, the orbitofrontal and posterior cingulate cortices, was associated in AD patients with anosognosia compared to healthy controls, suggesting a disconnection within and between the self-related and memory-related networks (i.e. default mode network; Perrotin et al., 2015). Decreased connectivity between the medial prefrontal cortex and the posterior hippocampus is also reported in MCI and early AD patients with poor performance during self-appraisal task execution (Ries et al., 2012). Although cortical regions of the default mode network have diminished functional connectivity with the medial prefrontal cortex, connectivity between the medial prefrontal cortex and the posterior cingulate cortex is not associated with anosognosia (Ries et al., 2012).

- **Summary**: In MCI patients, anosognosia is associated with reduced functional within-network connectivity between the precuneus and bilateral inferior parietal lobes, left posterior cingulate cortex, left orbitofrontal cortex and reduced functional connectivity between the right hippocampus and left medial temporal cortex and right fusiform gyrus. While in mild to moderate AD patients reduced functional connectivity within the default mode network and reduced connectivity between the hippocampus and insular cortex is associated with anosognosia. In mild to moderate AD, unawareness of memory deficits is correlated to attenuated within-network connectivity in the medial prefrontal cortex and proximal areas. Reduced between network connectivity among the orbitofrontal cortex and the middle temporal cortex, and between the posterior cingulate cortex and the middle temporal cortex is also observed in mild to moderate AD.

## Discussion

The neural correlates of impaired self-awareness have previously been reviewed focusing on the role of the mediotemporal lobe in neurodegenerative diseases (Chavoix & Insausti, 2017) and on the cortical midline structures and the default mode network in AD (Weiler et al., 2016). While a previous review focused on the cumulative evidence from structural and functional neuroimaging studies (Zamboni & Wilcock, 2011), the current review includes updated data and discusses anosognosia and unawareness of memory deficits from both a group comparative and correlational perspective. First, available neuroimaging evidence was reported by neuroimaging technique as each technique has its own advantages and limitations. Secondly, since the aim of this review was to identify the brain perfusion patterns, activation regions, and network connectivity characteristics that distinguish AD and MCI patients with anosognosia from healthy controls, and AD and MCI patients without anosognosia, the results were evaluated not only by stage of cognitive decline (i.e. MCI or early AD) but also by how unawareness of memory deficits or anosognosia was presented (i.e. dichotomization into two groups, with anosognosia or without anosognosia, and correlation of awareness to brain perfusion, metabolism, activation or connectivity). The secondary aim of this review was to compare brain activation patterns between AD and MCI patients with anosognosia and to identify regional brain activation differences between self-appraisal task execution and resting state. In this manner, self-related processing in AD might provide insights into the underpinnings of anosognosia.

### Anosognosia and Neuroimaging in AD

Study design plays a key role in how the observed outcome is interpreted in dementia patients with anosognosia. Case-control studies comparing MCI or AD patients with anosognosia (the cases) to MCI or AD patients without anosognosia (the controls) allow for a dichotomized comparison of awareness of memory deficits. This approach has the advantage of detecting specific regional differences in regard to the neural correlates of anosognosia, yet with limited capacity to assess awareness of memory deficits continuously. In contrast, studies that correlate awareness to indirect measures of neural activity (i.e. neuroimaging outcomes), rather than unawareness status, provide a continuous look into anosognosia. Nonetheless, with a limited proficiency to differentiate cases from controls.

#### Changes in Perfusion and Metabolism Related to Anosognosia

The frequency of anosognosia, as well as the degree of frontal lobe dysfunction through neuropsychological evaluation, increases with disease progression (Yoon et al., 2017). In support of these findings, the SPECT studies reviewed here have consistently reported decreased perfusion in the frontal lobes in AD patients with anosognosia compared to those without anosognosia (Hanyu et al., 2008; Starkstein et al., 1995; Sedaghat et al., 2010; Tagai et al., 2018), compared to healthy controls (Mimura & Yano, 2006), and when perfusion is correlated to unawareness (Derouesné et al., 1999; Reed et al., 1993; Shibata et al., 2008; Vogel et al., 2005). While brain perfusion SPECT studies primarily identify anosognosia in AD and amnestic MCI as a frontal lobe dysfunction, the18F fluorodeoxyglucose PET studies reviewed above show involvement of the cortical midline structures (Gerretsen et al., 2017; Nobili et al., 2010; Salmon et al., 2006; Therriault et al., 2018; Vannini et al., 2017) in the first stages of AD and dysfunction of the frontal lobe in later stages (Harwood et al., 2005; Jedidi et al., 2014; Sultzer et al., 2014). This pattern of disease progression parallels the histological changes described by Braak and Braak (1991) where the mediotemporal lobe is first affected, followed by the posterolateral cortical regions and affecting the frontal cortex later (Bokde, Ewers, & Hampel, 2009). Although SPECT studies on anosognosia show a predominance of frontal lobe dysfunction, differences among the brain perfusion areas associated with unawareness of memory deficit could be related to the AD population spectrum included and the diversity of anosognosia measurement instruments. Patients included in the reviewed SPECT studies range from amnestic MCI to moderate AD patients and while most studies used an anosognosia measurement instrument, a couple of studies depended exclusively on the examiner’s judgment. This is a validated screening method for anosognosia but not quantifiable as a self-appraisal instrument or task. Addressing the vascular component of brain perfusion differences in dementia patients, Starkstein and colleagues (Starkstein et al., 1996) report reduced perfusion in vascular dementia compared to AD patients. This finding highlights the association between increased frontal dysfunction and a vascular dementia pathology in some patients with anosognosia.

#### Changes in Activation and Connectivity Related to Anosognosia

Comparable to brain metabolism studies, reviewed activation and connectivity studies generally show decreased activation in and connectivity with the cortical midline structures, in particular, the medial prefrontal cortex and the posterior cingulate cortex associated with anosognosia in MCI and AD. The implementation of a self-appraisal task results in consistent hypoactivation (i.e. bilateral precuneus, bilateral hippocampus, orbitofrontal cortex and posterior cingulate cortex, medial frontal lobe, right inferior frontal gyrus, left inferior parietal lobule, left angular gyrus, and left superior temporal gyrus) in patients with anosognosia or impaired awareness of memory deficits. A spatial mentalizing gradient has been proposed by Denny, Kober, Wager, and Ochsner (2012), where self-related judgments activate the ventral medial prefrontal cortex and other-related judgments activate the dorsal medial prefrontal cortex. A functioning network involving the medial prefrontal and anterior temporal cortices is necessary for correct and updated personal information relating to the “petrified self” hypothesis (i.e. where memory impairment produces an aberrant personal information update) associated with self-awareness (Mograbi, Brown, & Morris, 2009; Morris & Mograbi, 2013). Furthermore, AD patients with anosognosia preserve the ability to judge others, adding to the interpretation of the medial prefrontal cortex as a key component of a neuronal system implicated in updating self-awareness (Zamboni et al., 2013). It has been previously recognized that the medial prefrontal and anterior temporal cortices are involved in self-awareness in evaluative processes, in self-judgment within social contexts, and in the long term assessment of the self (Zamboni et al., 2013).

Recently, incorporating other-related tasks has added value to the study of anosognosia. AD patients failed to activate the anterior temporal lobe during self-appraisal (Zamboni et al., 2013). While the anterior temporal lobes have been associated with semantic memory and conceptual knowledge, other theories identify these regions as an auxiliary of the social cognition system to support learning facts about others (Zamboni et al., 2013). The lateral middle temporal cortex network, middle hippocampal regions, and insular cortex have been linked with retrieval of personal memories, planning, episodic memory recall, episodic future thinking, mind wandering and episodic buffer for working memory (Berlingeri et al., 2015). Self-reference effect refers to the phenomenon that explains why, in healthy individuals, retrieval of information is more accurate when the information is encoded about the self, rather than related to other people (Symons & Johnson, 1997). Self-reference recollection effect alludes to recollection-based retrieval of information that has been previously associated with the self (Conway, Dewhurst, Pearson, & Sapute, 2001; Conway & Dewhurst, 1995). The self-reference effect and self-reference recollection effect provide a theoretical viewpoint attempting to explain the interaction between self-reference retrieval processes and memory within the Self-Memory System conceptual context.

The “petrified self” hypothesis proposes that memory impairment produces an aberrant personal information update (Mograbi et al., 2009; Morris & Mograbi, 2013). Northoff and colleagues propose a hierarchical framework of cortical regions related to the concept of self. In this framework, the sensory cortex is involved with sensory processing which belongs to the domain of the body called the “proto” or “bodily” self (Northoff et al., 2006; Northoff, Qin, & Feinberg, 2011). Self-referential processing is the cognitive process associated with bodily, mental or autobiographical self-related stimuli (Northoff et al., 2011). A dysfunction of the episodic and semantic memory retrieval process could be affecting the Self-Memory System. A possible mechanism could involve the sensory cortex, especially the anterior cortical midline structures, which mediate a bottom-up modulation of medial cortex activity, involved in self-referential processing. This dysfunction of the Self-reference effect and Self-reference recollection effect leads to failure to activate the core self during encoding of self-referential processing leading in turn to anosognosia of memory deficits (Northoff et al., 2006).

Coherence, a process during autobiographical recall where memories are reconfigured during retrieval to support our current beliefs and goals, is a principle that shapes memory construction (Conway, 2005; El Haj, Antoine, Nandrino, & Kapogiannis, 2015). The disruption of this episodic-autobiographical memory is another possible mechanism involved in the disruption of episodic and semantic memory as possible sources of anosognosia. The self, autonoetic consciousness and episodic-autobiographical memory are intimately related (Markowitsch & Staniloiu, 2011). Episodic-autobiographical memory is hypothesized to be the conjunction of subjective time, autonoetic consciousness and the experiencing of the self (Markowitsch & Staniloiu, 2011). Autonoetic consciousness reflects the capacity of continued existence, providing a sense of continuity and identity within a personal life history (Vandekerckhove & Panksepp, 2011). Autonoetic consciousness can be constituted of explicit self-awareness or the explicit awareness of something or someone in the past and future time-space contexts (Vandekerckhove & Panksepp, 2011). For further information regarding proposed theories describing the characterization of the underlying processes and the associated neuroanatomy on the recollection of episodic memory as a reconstructive process, planning and imagination, we refer to the review by Hassabis and Maguire (2009).

Self-related processing establishes the relation between organism and stimulus and alludes to stimuli that are experienced as strongly related to one’s own person, not to be confused with “insight”, which integrates cognitive and reflective functions (Northoff et al., 2006; Northoff et al., 2011). Higher order processing interacts with self-referential processing in the lateral prefrontal cortex, where the stimuli filtered by the self-referential processing are encoded (Northoff et al., 2006). This interaction between the self and memory processes seems altered in mild AD patients (Genon et al., 2014). A recent study investigating the association between scale-free dynamics of resting state fMRI activity and self-consciousness in healthy young individuals, reports a positive correlation between medial prefrontal cortex activity and self-consciousness (Huang, Obara, Davis 4th, Pokorny, & Northoff, 2016). Furthermore, Huang and colleagues (Huang et al., 2016) provide evidence of a direct relationship between resting state activity (i.e. spontaneous brain activity) and self-consciousness, providing evidence of the “rest-self” overlap in the medial prefrontal cortex (Huang et al., 2016). Increasing evidence associates self-relevant judgments to the ventral medial prefrontal cortex, while the dorsal medial prefrontal cortex seems to play a role in making judgments about the external world (Denny et al., 2012). Furthermore, decreased gray matter volume in the right ventrolateral prefrontal cortex, an area involved in generating and maintaining working memory, has been associated to reduced self-reflectiveness in patients with schizophrenia (Orfei, Piras, Macci, Caltagirone, & Spalletta, 2013). These structural changes highlight the role of the prefrontal cortex in self-referential processes beyond neurodegenerative disorders. Greater activation in the angular gyrus was found in controls compared to a group of early AD and MCI patients performing a self-referential processing judgment task, suggesting that the greater the activity the better the recognition of self-processed items (Gaubert et al., 2017).

Changes in activation and connectivity have also been recently studied in MCI with anosognosia. A meta-analysis of MCI patients and patients with subjective cognitive complaints showed that MCI patients have knowledge of their neuropsychological deficits and that their level of awareness is linked to several cognitive capacities. Interestingly, an association between awareness and neuropsychological functioning is present only after a certain threshold of cognitive deterioration is reached (Piras et al., 2016). The conjunction analysis of a visual episodic recognition task and an autobiographical self-appraisal task revealed that the posterior cingulate cortex is the sole region active during both tasks in healthy older adults, while only activation of the posterior cingulate cortex in the self-appraisal task is observed in MCI patients, suggesting functional deterioration during episodic retrieval (Ries et al., 2006). Current theories of the role of the posterior cingulate cortex in cognitive function include modulation of arousal and awareness, controlling internally directed thought, mediation between internal and external attention and detection in environmental changes (Leech & Sharp, 2014). The dorsal posterior cingulate cortex also shows strong connectivity with the default mode network and, due to its complex interactions with other intrinsic connectivity networks such as the left frontoparietal control network also share connectivity with parts of the dorsal attention network, a sensorimotor network and a salience network serving as a key node and network hub for cognitive function (Leech & Sharp, 2014).

#### Neural Correlates in Anosognosia and Associated with Unawareness of Memory Deficit in MCI are AD Patients

**Fig. 2:**
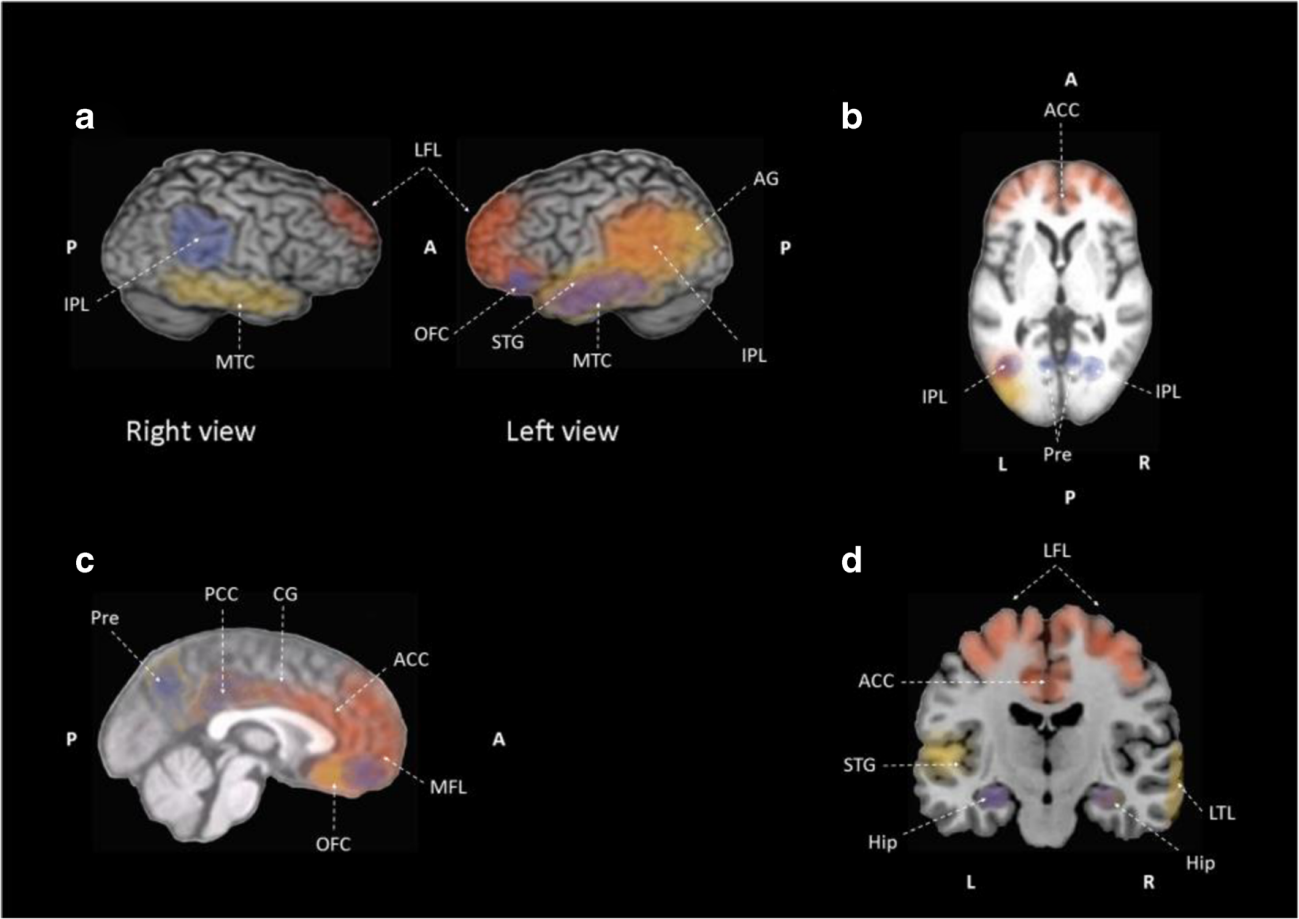
Graphical representation of the neural correlates of anosognosia, case-control studies, in mild cognitive impairment patients based on data from: Hanyu et al., 2008, Nobili et al., 2010, Therriault et al., 2018, Vannini et al., 2017. Hypoperfusion in the bilateral lateral (LFL) and medial frontal (MFL) lobes, the bilateral anterior cingulate cortex (ACC) and cingulate gyri (CG), and the left inferior parietal region (IPL). Hypometabolism of the posterior cingulate cortex, precuneus (Pre), right hippocampus (Hip), bilateral temporal cortex (MTC), left inferior parietal lobule (IPL), the left angular gyrus (AG), and the left superior temporal gyrus (STG). Reduced functional within-network connectivity between precuneus (Pre) and bilateral inferior parietal lobes (IPL), left posterior cingulate cortex, left orbitofrontal cortex (OFC). Reduced functional connectivity between right hippocampus (Hip) and left medial temporal cortex (MTC) and right fusiform gyrus (not shown). **a** lateral view, **b** axial view, **c** sagittal view, and **d** coronal view. Hypoperfusion in red, hypometabolism in orange, reduced within-network connectivity in blue, and reduced between network connectivity in purple

**Fig. 3:**
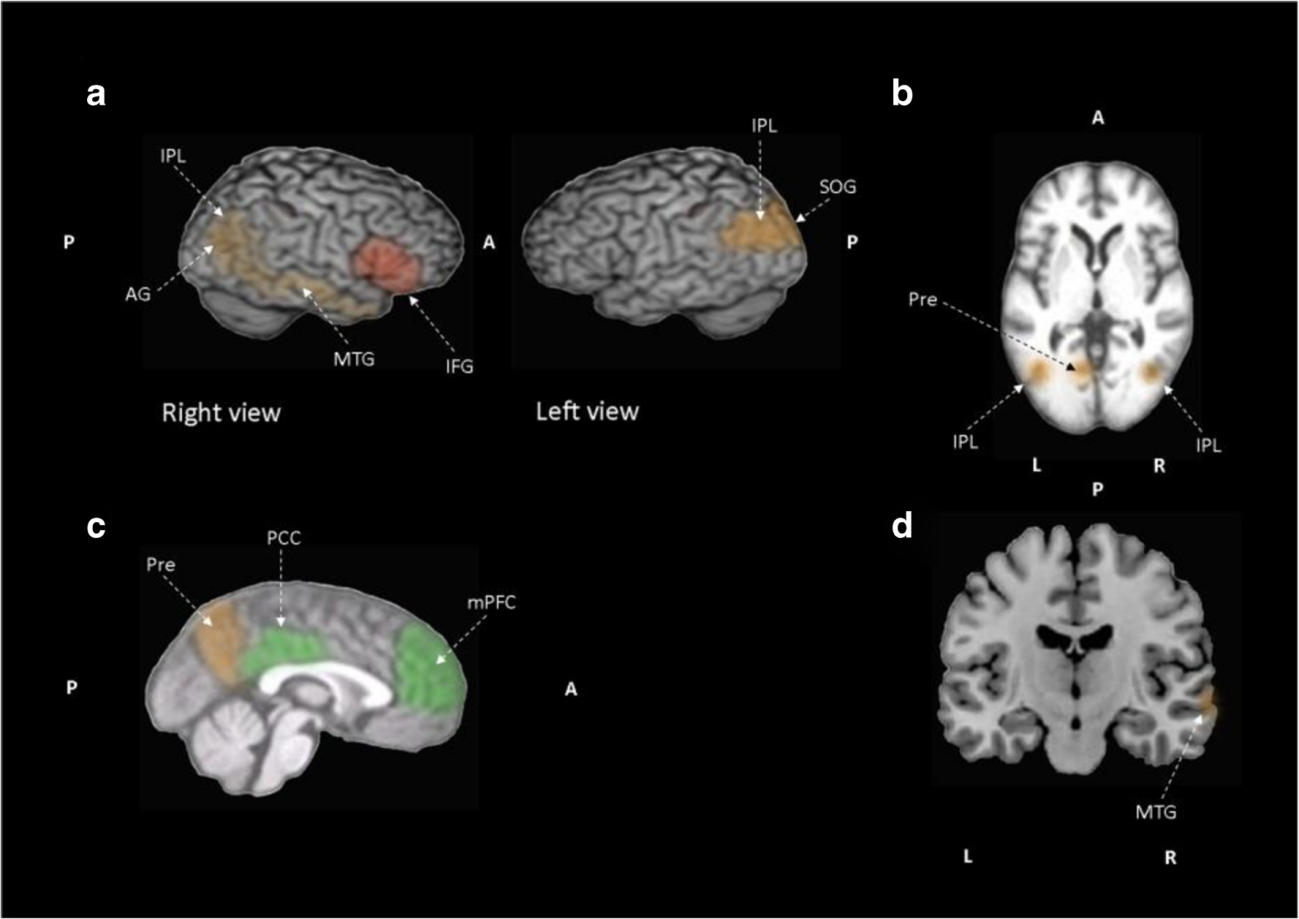
Graphical representation of the neural correlates of unawareness of memory deficits in mild cognitive impairment based on data from: Nobili et al., 2010, Ries et al., 2007, Vogel et al., 2005. Hypoperfusion in the right inferior frontal gyrus (IFG). Hypometabolism in the left precuneus (Pre), left inferior parietal lobe (IPL) and left superior occipital gyrus (SOG), right inferior parietal lobe (IPL), right angular gyrus (AG) and right middle temporal gyrus (MTG). Hypoactivation in the bilateral medial prefrontal (mPFC) and posterior cingulate cortices (PCC). **a** lateral view, **b** axial view, **c** sagittal view, and **d** coronal view. Hypoperfusion in red, hypometabolism in orange, and hypoactivation in green

**Fig. 4:**
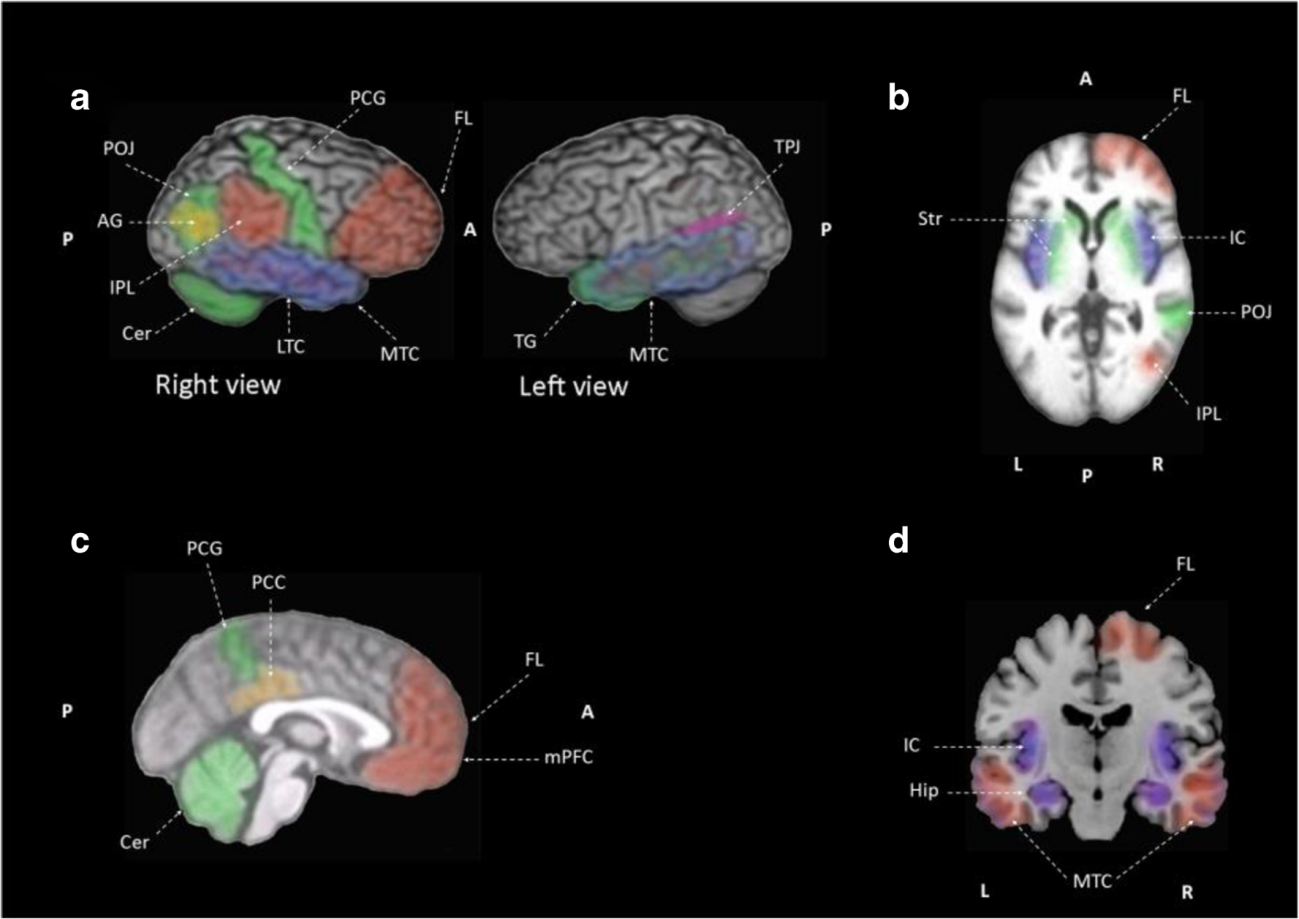
Graphical representation of the neural correlates of anosognosia, case-control studies, in mild to moderate AD patients based on data from: Amanzio et al., 2011, Berlingeri et al., 2015, Gerretsen et al., 2017, Sedaghat et al., 2010, Starkstein et al., 1995, Tagai et al., 2018. Hypoperfusion in the right frontal lobe (FL, frontal inferior, frontal superior and prefrontal cortex), the right inferior parietal (IPL), bilateral medial temporal cortex (MTC), and right prefrontal cortex. Hyperperfusion of left temporoparietal junction (TPJ). Hypometabolism in posterior cingulate cortex and right angular gyrus (AG). Hypoactivation in the right postcentral gyrus (PCG), right parietotemporal and parietooccipital junction (POJ) and the left temporal gyrus (TG), striatum (Str) and cerebellum (Cer). Reduced functional connectivity within the default mode network (comprised of the lateral temporal cortex, LTC, the hippocampus, Hip, and the insula, IC), and reduced connectivity between the hippocampus and insular cortex. **a** lateral view, **b** axial view, **c** sagittal view, and **d** coronal view. Hypoperfusion in red, hyperperfusion in pink, hypoactivation in green, reduced within-network connectivity in blue, and reduced between network connectivity in purple

**Fig. 5:**
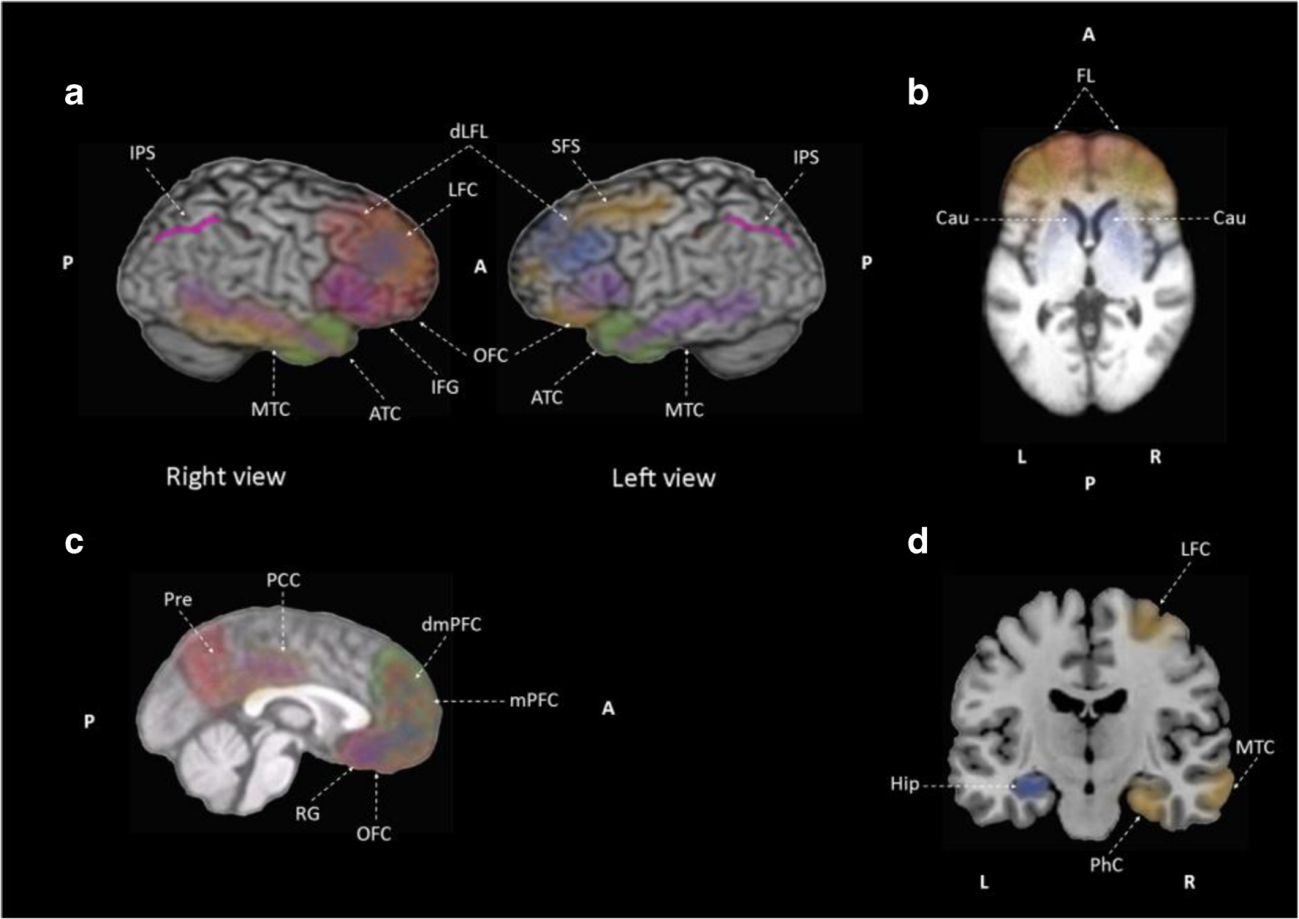
Graphical representation of the neural correlates of unawareness of memory deficits in mild to moderate AD based on data from: Derouesné et al., 1999, Harwood et al., 2005, Jedidi et al., 2014, Mimura & Yano, 2006, Perrotin et al., 2015, Reed et al., 1993, Ries et al., 2012, Ruby et al., 2009, Salmon et al., 2006, Shibata et al., 2008, Sultzer et al., 2014, Zamboni et al., 2013. Hypoperfusion of bilateral frontal regions (FL, medial frontal, medial prefrontal cortex, mPFC, and orbitofrontal cortex, OFC), the right dorsolateral frontal lobe (dLFL), the right precuneus (Pre) and right inferior frontal gyrus (IFG). Hypometabolism of the bilateral medial prefrontal cortex, bilateral orbitofrontal cortex and posterior cingulate cortices, the right lateral frontal cortex (LFC), the right parahippocampal cortex (PhC), the right gyrus rectus (GR), the right middle temporal cortex (MTC), left superior frontal sulcus (SFS), left dorsomedial prefrontal cortex. Hypoactivation of the bilateral dorsomedial prefrontal cortex, bilateral medial prefrontal cortex and bilateral anterior temporal cortices (ATC). Attenuated within-network functional connectivity in the medial prefrontal cortex and proximal areas [bilateral dorsolateral prefrontal cortex (dLFL), bilateral caudate (Cau), and left posterior hippocampus (Hip)]. Hyperactivation of intraparietal sulcus (IPS). Reduced between network connectivity among the orbitofrontal cortex and the middle temporal cortex, and between the posterior cingulate cortex and the middle temporal cortex. **a** lateral view, **b** axial view, **c** sagittal view, and **d** coronal view. Hypoperfusion in red, hypometabolism in orange, hypoactivation in green, hyperactivation in pink, reduced within-network connectivity in blue, and reduced between network connectivity in purple

Two distinct designs characterized the studies in this review, namely, those which compared brain activity (i.e. perfusion, metabolism, activation, and connectivity) in subjects with or without anosognosia and those which correlated awareness to brain activity. To summarize the results, a graphical representation of the neural correlates in MCI (Fig. 2) and AD (Fig. 4) patients with anosognosia was created with the assistance of the IMAIOS brain atlas of human anatomy with MRI (IMAIOS SAS, Montpellier, France) using the brain regions, brain coordinates or Brodmann areas extracted from reviewed articles. For the articles that correlated impaired awareness to brain activity, graphical representations of the neural correlates of unawareness of memory deficits in MCI (Fig. 3) and AD (Fig. 5) patients were created. The studies that compare MCI patients with and without anosognosia report hypoperfusion of the lateral and medial frontal lobes, the anterior cingulate and cingulate gyri in both hemispheres, and the left inferior parietal region (Fig. 2). These studies also report hypoactivation of the left inferior parietal lobule, left angular gyrus and left superior temporal gyrus (Fig. 2). The left inferior parietal region is the region of overlap in SPECT and PET studies included in this review. The studies that correlate awareness to brain activity in MCI patients contrast with studies comparing MCI patients with and without anosognosia. Studies that correlate awareness to brain activity in this patient group report hypoperfusion in the right inferior frontal gyrus, while also reporting hypometabolism in the left precuneus, left inferior parietal lobe, left superior occipital gyrus, right inferior parietal lobe, right angular gyrus and right middle temporal gyrus (Fig. 3). In addition, these studies report hypoactivation in the medial prefrontal cortex and posterior cingulate cortex in both hemispheres (Fig. 3).

While data concerning MCI patients is limited, more studies have investigated the neural correlates of anosognosia and unawareness in AD patients. Comparisons between mild to moderate AD patients with anosognosia and AD patients without anosognosia report hypoperfusion in the right frontal lobe (frontal inferior, frontal superior, and prefrontal cortex), the right inferior parietal lobe, and bilateral medial temporal cortex (Fig. 4). At the same time, hypoactivation in the right postcentral gyrus, right parietotemporal and parietooccipital junction, and the left temporal gyrus, striatum, and cerebellum has also been reported in AD patients with anosognosia compared to AD patients without anosognosia (Fig. 4). Finally, reduced within-network functional connectivity in the default mode network (i.e. comprised of the lateral temporal cortex, the hippocampus, and the insula) and reduced between network connectivity between the hippocampus and insular cortex have been reported in AD patients with anosognosia when compared to cohorts without unawareness of memory deficits (Fig. 4). The studies that correlate unawareness to brain activity in AD patients report hypoperfusion of bilateral frontal regions (medial frontal and orbitofrontal cortex), the right dorsolateral frontal lobe, the right precuneus, and inferior frontal gyrus as unawareness of memory deficits progresses (Fig. 5). Hypometabolism of the bilateral medial frontal cortex, bilateral orbitofrontal and posterior cingulate cortices, the right lateral frontal cortex, the right parahippocampal cortex, the right gyrus rectus, the right middle temporal cortex, left superior frontal sulcus and left dorsomedial prefrontal cortex has also been related to reduced awareness (Fig. 5). Functional MRI studies correlating awareness and brain activity report hypoactivation of the bilateral dorsomedial prefrontal cortex, bilateral medial prefrontal and anterior temporal cortices in unawareness (Fig. 5). Regarding connectivity, attenuated within-network connectivity in the medial prefrontal cortex and proximal areas (bilateral dorsolateral prefrontal cortex, bilateral caudate, and left posterior hippocampus) was related to unawareness of memory deficits. Similarly, reduced connectivity between the orbitofrontal and the medial temporal cortices, and between the posterior cingulate cortex and the medial temporal cortex has been associated with unawareness in AD patients (Fig. 5). To summarize, among the regions that share changes in brain activity across functional neuroimaging techniques and study design as used in the studies reviewed here, are the bilateral temporal lobe, right frontal lobe, right medial prefrontal cortex, and right orbitofrontal cortex. Changes in within-network connectivity generally concern regions pertaining to the default mode network for both anosognosia group comparisons as well as correlation analysis of unawareness of memory deficits in AD patients.

#### The Possible Role of Cortical Midline Structures in Anosognosia

Cconsidering that the main components of anosognosia in early stages of AD might be associated with the failure of memory management of incoming information and self-assessment (Avondino & Antoine, 2016), the cortical midline structures (i.e. medial prefrontal cortex, anterior cingulate cortex and posterior cingulate cortex) along with other brain regions part of the default mode network functional hubs (i.e. precuneus and angular gyrus) should have disrupted neuronal activity (e.g. metabolism and connectivity). The cortical midline structures are part of the self-referential processing and autobiographical-self network in healthy subjects and subjects between the ages of 18 and 50 (Northoff et al., 2006; Araujo, Kaplan, & Damasio, 2013). The medial prefrontal cortex, posterior cingulate cortex, and inferior parietal lobule have been proposed as the core-self regions of the default mode network, with the posterior cingulate cortex and inferior parietal lobule driving the self-related processes and the medial prefrontal cortex having regulatory gateway function (Davey, Pujol, & Harrison, 2016). Strong overlap between stimulus induced activity in the cortical midline structures during self-referential processing and resting state activity in the default mode network exists (Northoff et al., 2011). A study directly comparing brain regions activated by explicit self-reference during judgment of self-trait adjectives and rest conditions relative to a semantic task without self-reference attempts to discern this overlap (Whitfield-Gabrieli et al., 2011). Explicit self-reference activated the dorsal medial prefrontal cortex, while the rest condition engaged the precuneus. Meanwhile, both self-reference and rest engaged the ventral medial prefrontal cortex and posterior cingulate cortex (Whitfield-Gabrieli et al., 2011). Healthy older adults, MCI, and early AD patients also have activation of this self-referential processing and the autobiographical-self network (Gaubert et al., 2017). Few studies have explored the association between autobiographical memory, self-knowledge, and anosognosia in AD, with judgment of self-relevant information tasks attempt to shed light on this topic (Morris & Mograbi, 2013). Mild AD patients have poor retrieval of self-related and other-related items compared to healthy subjects, represented by the correlation between retrieval performance and functional brain activity (Genon et al., 2014). Overall, we expect that activation patterns during self-appraisal for patients with anosognosia will also differ from patients without anosognosia, affecting regions previously associated with self-awareness, such as the cortical midline structures and other default mode network regions, earlier in MCI patients than AD patients with anosognosia.

### From Neural Correlates to Network Architecture

The perfusion, metabolism and activation changes seen in MCI and AD patients with anosognosia and unaware of memory deficits, demonstrate a characteristic pattern. This pattern suggests the involvement of the cortical midline structures during initial stages of cognitive decline, reduced activity of parietotemporal structures in later stages and culminating in a frontotemporal dysfunction. This pattern follows a posterior to ventral along with an anterior to dorsal gradient, initiating at the posterior cingulate cortex, precuneus and angular gyrus progressing to the anterior cingulate and medial prefrontal cortices and ending with mediotemporal lobe and inferior parietal lobule involvement. To understand the differences from the expected outcome and finding from this review one must consider the population (i.e. AD or MCI, with or without anosognosia), the indirect measures of neural activity, and the study design. Based on the limited literature concerning the neural correlates measured in MCI patients with anosognosia (Fig. 2), reduced indirect neural activity is found in both cortical midline structures and parietotemporal structures. However, when studies correlating unawareness to indirect measures of neural activity (Fig. 3) are used to complement the case-control studies, the cortical midline structures (i.e. medial prefrontal cortex, anterior cingulate cortex and posterior cingulate cortex) but also other anatomical regions associated with the default mode network (i.e. precuneus, angular gyrus) show reduced indirect neural activity. Conventionally, the default mode network is studied through fMRI activation and connectivity studies. However, due to the insufficient fMRI evidence, perfusion and metabolic changes associated with the anatomical areas that comprise this network can be included as preliminary evidence. Regardless of the study design in the early AD population, the regions primarily affected at this stage of the cognitive decline continuum include the entire frontal lobe, mediotemporal lobe and the parietotemporal junction (Figs. 4 and 5) in addition to the regions affected in MCI.

#### Neural Correlates of Self-Appraisal

Recently, attempts to underpin the brain regions related to the “self” have led to the implementation of self-appraisal task fMRI. Understanding the cognitive mechanisms and neural correlates of self-appraisal can provide insight into the topological network changes across the cognitive decline continuum. Hypoactivation of the medial prefrontal cortex and posterior cingulate cortex is reported in MCI against controls, evaluated for anosognosia with a self–appraisal task (Ries et al., 2007). While more fMRI evidence is available in AD patients with impaired awareness of memory deficits, only two activation and connectivity case-control studies studying anosognosia (Fig. 4) and four correlating awareness to activation and connectivity (Fig. 5) were available at the time of this review. The results presented in the self-appraisal studies suggest decreased activation of the medial prefrontal cortex, anterior temporal cortex, and intraparietal sulcus (Ruby et al., 2009; Zamboni et al., 2013). Although the current evidence suggests differences in activation between AD or MCI and healthy controls, more evidence is needed exploring the differences between MCI and AD patients with anosognosia using self-appraisal task fMRI. Both indirect measures of neural activity (i.e. hemodynamic and metabolic) show changes in the presence of anosognosia, regardless of the way the phenomenon of unawareness of memory deficits is observed (i.e. study design). However, these neuroimaging techniques strongly depend on vascular factors that might account for regional localization differences. This review presents evidence from different neuroimaging techniques (i.e. SPECT, PET, and fMRI) but the results must be interpreted independently by the technique to correctly study the phenomenon of anosognosia in MCI and AD.

One way to explain the heterogeneity and overlap of the regions involved in anosognosia in different stages of cognitive decline is to understand that hemodynamic and metabolic neuroimaging biomarkers both are compromised by vascular dysregulation. Vascular dysregulation has been proposed as the initial pathologic event leading to late-onset AD with the multifactorial causal model of brain (dis) organization recently contesting the most cited model (i.e. hypothetical model of dynamic biomarkers) which tries to explain the temporal appearance of pathophysiological biomarkers associated to AD (Iturria-Medina et al., 2017). While the hypothetical model of dynamic biomarkers (Jack et al., 2013) introduces a possible temporal biomarker incidence model, it fails to reflect the multifactorial and interactive nature of biomarkers with different spatiotemporal scales (Iturria-Medina et al., 2016).

#### Network Dysfunction

Effective connectivity refers explicitly to the influence that one neural system exerts over another. Functional MRI allows for the assessment of effective connectivity, which is associated to a model of interactions or coupling (Friston, 2011). The cerebral disconnection syndrome hypothesis suggests that AD behavioral pathology is due to disturbances of the brain’s effective connectivity, thereby affecting any task involving communication between brain regions (Delbeuck, Van der Linden, & Collette, 2003; Vallet, Hudon, Simard, & Versace, 2013). Bokde, Ewers and Hampel (2009, p. 126), describe the disconnection syndrome hypothesis as “a disruption in the temporal-spatially coordinated activity among different regions in the brain rather than isolated changes in particular brain regions [which] may underlie cognitive impairment in AD”. A few studies have attempted to test the disconnection syndrome hypothesis in AD using visual and auditory cross-modal effects, reporting impaired cross-modal integration during perceptual priming, hence supporting the disconnection syndrome hypothesis (Delbeuck, Collette, & Van der Linden, 2007; Vallet et al., 2013). The disconnection syndrome hypothesis (Delbeuck et al., 2007; Vallet et al., 2013) associated with the cascading network failure model could explain the connectivity changes seen throughout the cognitive decline continuum. The disconnection syndrome hypothesis suggests a coordinated activity disruption among different brain regions. However, the source (i.e. hemodynamic, metabolic or electrical) of these temporal-spatial correlational differences is currently under investigation. The cascading network failure model alludes to the disconnection effect proliferating to downstream nodes eventually leading to failure of the entire system (Jones et al., 2016).

### General Limitations

Anosognosia is a heterogeneous condition with more than one recognized etiology. A conceptual model of anosognosia based on the results presented in this review is not possible due to the heterogeneity of measures to assess awareness of memory deficits, impaired insight, and impaired self-appraisal. This heterogeneity of measures is the greatest limitation of this review, as not all selected studies focus on anosognosia for memory impairment. The interpretation of results presented in this review, regarding unawareness of memory deficits as a phenomenon relating to anosognosia, is also limited due to the heterogeneity of the methods used to assess anosognosia, as well as possible misclassification in the studies which dichotomize the population and the accuracy of the awareness staging in the studies which employ an awareness index. Given the vast quantity of awareness or unawareness measurement instruments, standardization and cross-validation of these instruments for the prodromal and clinical AD are needed.

Another limitation of this review concerns the distinction between the neural correlates of anosognosia and global disease progression. More functional neuroimaging studies employing memory tasks underpinning the role of attention and inhibition in patients with different stages of AD with anosognosia are thus needed. While absolute activity levels are not assessed by SPECT, as perfusion ratio measurements quantify relative perfusion (Reed et al., 1993), the resting state paradigm, even in patients with dementia and anosognosia, fundamentally incorporates self-referential stimuli since it requires the subject to consciously or unconsciously be aware of body movement. Concurrently, the process of being scanned is a self-evaluative task, hence limiting self-reference assessment. In this review, insufficient evidence was found investigating the role of executive function deficits in AD with anosognosia. Deficits in attention and inhibition could be involved in the autobiographical decline in AD (El Haj et al., 2015). Perfusion SPECT studies primarily identify anosognosia as a frontal lobe dysfunction, while PET studies report frontal lobe involvement as AD progresses. If attention is a frontal lobe function, attentional deficits in AD may contribute to autobiographical memory dysfunction (El Haj et al., 2015). Furthermore, attentional deficits could be a confounding factor in functional neuroimaging studies. Small sample sizes limit multivariate regression models which are often used by PET studies included in this review.

Taking into consideration the limited evidence, the cerebral disconnection syndrome hypothesis and cascading network failure model can provide the conceptual framework to understand the functional neural correlates and topological network changes observed across the cognitive decline continuum. The current authors do not contend that unawareness of memory deficits results from a cerebral disconnection syndrome, as not all patients with anosognosia show aberrant communication between the cortical midline structures and the middle temporal regions. However, the brain regions affected in anosognosia of memory deficits show that regions associated with the self, memory retrieval and self-referential judgment are affected. In the case of anosognosia associated with AD and MCI, the disconnection syndrome hypothesis could explain functional connectivity changes, while the cascading network failure model provides a model of effective connectivity disruption associated with cognitive decline. However, at the moment there is insufficient evidence to make such assertions. Longitudinal studies evaluating self-awareness of memory deficits are necessary in order to evaluate whether anosognosia has an influence on disease progression from MCI to AD. Such studies would optimally implement appropriate diagnostic biomarkers or postmortem disease confirmation. Without longitudinal studies, the MCI cases included could be due to other etiologies (e.g. psychiatric, vascular or neurodegenerative) or even reflect the lack of diagnostic accuracy associated with a measurement error of neuropsychological tests. To explore causality between anosognosia and the order in which brain regions are affected, better designed connectivity studies comparing patients with and without anosognosia throughout the cognitive decline continuum are necessary. Better designed studies are also need to explore the association between unawareness of memory deficits and the disruption of brain regions involved in memory.

### Conclusion

We found that the neural correlates of anosognosia and unawareness of memory deficits in patients with MCI and AD differ depending on the imaging technique. Brain perfusion SPECT studies primarily associate anosognosia in MCI and AD patients with frontal lobe dysfunction. In contrast, AD patients with anosognosia show cortical midline hypometabolism and frontotemporal dysfunction assessed though 18F fluorodeoxyglucose PET metabolism and fMRI activation and AD patients with anosognosia exhibit reduced within-network connectivity in the default mode network (i.e. the lateral temporal cortex, the hippocampus, and the insular cortex). In AD patients, attenuated within-network functional connectivity is associated with the decline of awareness of memory deficits in the lateral middle temporal cortex network. Furthermore, AD patients with anosognosia have reduced connectivity between the hippocampus and insular cortex, while unawareness of memory deficits is correlated with reduced between-network connectivity among the orbitofrontal and the medial temporal cortices, and among the posterior cingulate and the medial temporal cortices.

## Electronic supplementary material

ESM 1 (DOCX 310 kb)

## References

[CR1] Albert, M. S., DeKosky, S. T., Dickson, D., Dubois, B., Feldman, H. H., Fox, N. C., … Phelps, C. H. (2011). The diagnosis of mild cognitive impairment due to Alzheimer's disease: Recommendations from the National Institute on Aging-Alzheimer's Association workgroups on diagnostic guidelines for Alzheimer's disease. *Alzheimer’s & Dementia, 7*(3), 270–279. 10.1016/j.jalz.2011.03.008. 10.1016/j.jalz.2011.03.008 PMC3312027 21514249

[CR2] Amanzio M., Torta, D. M., Sacco, K., Cauda, F., D'Agata, F., Duca, S., … Geminiani, G. C. (2011). Unawareness of deficits in Alzheimer's disease: Role of the cingulate cortex. *Brain, 134*(Pt 4), 1061–1076. 10.1093/brain/awr020. 10.1093/brain/awr020 21385751

[CR3] Araujo HF, Kaplan J, Damasio A (2013). Cortical midline structures and autobiographical-self processes: An activation-likelihood estimation meta-analysis. Frontiers in Human Neuroscience.

[CR4] Avondino E, Antoine P (2016). Heterogeneity of cognitive Anosognosia and its variation with the severity of dementia in patients with Alzheimer's disease. Journal of Alzheimer's Disease.

[CR5] Berlingeri M, Ravasio A, Cranna S, Basilico S, Sberna M, Bottini G, Paulesu E (2015). Unrealistic representations of "the self": A cognitive neuroscience assessment of anosognosia for memory deficit. Consciousness and Cognition.

[CR6] Bokde AL, Ewers M, Hampel H (2009). Assessing neuronal networks: Understanding Alzheimer's disease. Progress in Neurobiology.

[CR7] Braak H, Braak E (1991). Neuropathological stageing of Alzheimer-related changes. Acta Neuropathologica.

[CR8] Castrillo-Sanz, A., Andrés-Calvo, M., Repiso-Gento, I., Izquierdo-Delgado, E., Gutiérrez-Ríos, R., Rodríguez-Herrero, R., … Tola-Arribas, M. A. (2016). Anosognosia in Alzheimer disease: Prevalence, associated factors, and influence on disease progression. *Neurologia, 31*(5), 296–304. 10.1016/j.nrl.2015.03.006. 10.1016/j.nrl.2015.03.006 25976940

[CR9] Chavoix C, Insausti R (2017). Self-awareness and the medial temporal lobe in neurodegenerative diseases. Neuroscience and Biobehavioral Reviews.

[CR10] Clare, L., Nelis, S. M., Martyr, A., Roberts, J., Whitaker, C. J., Markova, I. S., … Morris, R. G. (2012). The influence of psychological, social and contextual factors on the expression and measurement of awareness in early-stage dementia: Testing a biopsychosocial model. *International Journal of Geriatric Psychiatry, 27*(2), 167–177. 10.1002/gps.2705 10.1002/gps.2705 21425345

[CR11] Clare L, Wilson BA, Carter G, Roth I, Hodges JR (2002). Assessing awareness in early-stage Alzheimer’s disease: Development and piloting of the memory awareness rating scale. Neuropsychological Rehabilitation.

[CR12] Conway MA (2005). Memory and the self. Journal of Memory and Language.

[CR13] Conway MA, Dewhurst SA (1995). The self and recollective experience. Applied Cognitive Psychology.

[CR14] Conway MA, Dewhurst SA, Pearson N, Sapute A (2001). The self and recollection reconsidered: How a ‘failure to replicate’ failed and why trace strength accounts of recollection are untenable. Applied Cognitive Psychology.

[CR15] Crook TH, Feher EP, Larrabee GJ (1992). Assessment of memory complaint in age-associated memory impairment: The MAC-Q. International Psychogeriatrics.

[CR16] Dalla Barba G, Parlato V, Iavarone A, Boller F (1995). Anosognosia, intrusions and 'frontal' functions in Alzheimer's disease and depression. Neuropsychologia.

[CR17] Davey CG, Pujol J, Harrison BJ (2016). Mapping the self in the brain's default mode network. Neuroimage.

[CR18] Davies M, Davies AA, Coltheart M (2005). Anosognosia and the two-factor theory of delusions. Mind & Language.

[CR19] De Carolis, A., Cipollini, V., Corigliano, V., Comparelli, A., Sepe-Monti, M., Orzi, F., …, Giubilei, F. (2015). Anosognosia in people with cognitive impairment: Association with cognitive deficits and behavioral disturbances. *Dementia and Geriatric Cognitive Disorders Extra, 5*(1), 42–50. 10.1159/000367987. 10.1159/000367987 PMC4361910 25852731

[CR20] Delbeuck X, Collette F, Van der Linden M (2007). Is Alzheimer's disease a disconnection syndrome? Evidence from a crossmodal audio-visual illusory experiment. Neuropsychologia.

[CR21] Delbeuck X, Van der Linden M, Collette F (2003). Alzheimer's disease as a disconnection syndrome?. Neuropsychology Review.

[CR22] Dennis EL, Thompson PM (2014). Functional brain connectivity using fMRI in aging and Alzheimer's disease. Neuropsychology Review.

[CR23] Denny BT, Kober H, Wager TD, Ochsner KN (2012). A meta-analysis of functional neuroimaging studies of self- and other judgments reveals a spatial gradient for mentalizing in medial prefrontal cortex. Journal of Cognitive Neuroscience.

[CR24] Derouesné C, Thibault S, Lagha-Pierucci S, Baudouin-Madec V, Ancri D, Lacomblez L (1999). Decreased awareness of cognitive deficits in patients with mild dementia of the Alzheimer type. International Journal of Geriatric Psychiatry.

[CR25] El Haj M, Antoine P, Nandrino JL, Kapogiannis D (2015). Autobiographical memory decline in Alzheimer's disease, a theoretical and clinical overview. Ageing Research Reviews.

[CR26] Franzmeier, N., Caballero, M. Á. A., Taylor A. N. W., Simon-Vermot, L., Buerger, K., Ertl-Wagner, B., … Ewers M, Alzheimer’s Disease Neuroimaging Initiative. ( 2017). Resting-state global functional connectivity as a biomarker of cognitive reserve in mild cognitive impairment. *Brain Imaging and Behavior, 11*(2), 368–382. 10.1007/s11682-016-9599-1. 10.1007/s11682-016-9599-1 27709513

[CR27] Friston KJ (2011). Functional and effective connectivity: A review. Brain Connectivity.

[CR28] Gaubert, M., Villain, N., Landeau, B., Mézenge, F., Egret, S., Perrotin, A., … G. (2017). Neural correlates of self-reference effect in early Alzheimer's disease. *Journal of Alzheimer's Disease, 56*(2), 717–731. 10.3233/JAD-160561 10.3233/JAD-160561 28035923

[CR29] Genon, S., Bahri, M. A., Collette, F., Angel, L., d'Argembeau, A., Clarys, D., … Bastin, C. (2014). Cognitive and neuroimaging evidence of impaired interaction between self and memory in Alzheimer's disease. *Cortex, 51*, 11–24. 10.1016/j.cortex.2013.06.009 10.1016/j.cortex.2013.06.009 23993283

[CR30] Gerretsen, P., Chung, J. K., Shah, P., Plitman, E., Iwata, Y., Caravaggio, F., … Graff-Guerrero, A., & Alzheimer’s Disease Neuroimaging Initiative. (2017). Anosognosia is an independent predictor of conversion from mild cognitive impairment to Alzheimer's disease and is associated with reduced brain metabolism. *Journal of Clinical Psychiatry, 78*(9), e1187-e1196. 10.4088/JCP.16m11367. 10.4088/JCP.16m11367 29022655

[CR31] Hafkemeijer, A., Möller, C., Dopper, E.G., Jiskoot, L.C., van den Berg-Huysmans, A. A., van Swieten, J. C., ... Rombouts, S. A. (2017). A longitudinal study on resting state functional connectivity in behavioral variant frontotemporal dementia and Alzheimer's disease. *Journal of Alzheimer’s Disease, 55*(2), 521–37. 10.3233/JAD-150695. 10.3233/JAD-150695 27662284

[CR32] Hanyu, H., Sato, T., Akai, T., Shimizu, S., Hirao, K., Kanetaka, H., Iwamoto, T., … Koizumi, K. (2008). Neuroanatomical correlates of unawareness of memory deficits in early Alzheimer's disease. *Dementia and Geriatric Cognitive Disorders, 25*(4), 347–353. 10.1159/000119594. 10.1159/000119594 18319600

[CR33] Harwood DG, Sultzer DL, Feil D, Monserratt L, Freedman E, Mandelkern MA (2005). Frontal lobe hypometabolism and impaired insight in Alzheimer disease. The American Journal of Geriatric Psychiatry.

[CR34] Harwood DG, Sultzer DL, Wheatley MV (2000). Impaired insight in Alzheimer disease: Association with cognitive deficits, psychiatric symptoms, and behavioral disturbances. Neuropsychiatry, Neuropsychology, and Behavioral Neurology.

[CR35] Hassabis D, Maguire EA (2009). The construction system of the brain. Philosophical transactions of the Royal Society of London. Series B, biological sciences.

[CR36] Huang Z, Obara N, Davis HH, Pokorny J, Northoff G (2016). The temporal structure of resting-state brain activity in the medial prefrontal cortex predicts self-consciousness. Neuropsychologia.

[CR37] Iturria-Medina Y, Carbonell FM, Sotero RC, Chouinard-Decorte F, Evans AC, Alzheimer's Disease Neuroimaging Initiative (2017). Multifactorial causal model of brain (dis)organization and therapeutic intervention: Application to Alzheimer's disease. Neuroimage.

[CR38] Iturria-Medina, Y., Sotero, R.C., Toussaint, P.J., Mateos-Pérez, J.M., Evans, A.C., & Alzheimer’s Disease Neuroimaging Initiative. (2016). Early role of vascular dysregulation on late-onset Alzheimer's disease based on multifactorial data-driven analysis. *Nature Communications, 7*, 11934. 10.1038/ncomms11934. 10.1038/ncomms11934 PMC4919512 27327500

[CR39] Jack, C. R., Knopman, D. S., Jagust, W. J., Petersen, R. C., Weiner, M. W., Aisen, P. S., … Trojanowski, J. Q. (2013). Tracking pathophysiological processes in Alzheimer's disease: An updated hypothetical model of dynamic biomarkers. *Lancet Neurology, 12*(2) 207–216. 10.1016/S1474-4422(12)70291-0. 10.1016/S1474-4422(12)70291-0 PMC3622225 23332364

[CR40] Jedidi, H., Feyers, D., Collette, F., Bahri, M. A., Jaspar, M., d'Argembeau, A., … Bastin, C. (2014). Dorsomedial prefrontal metabolism and unawareness of current characteristics of personality traits in Alzheimer's disease. *Social Cognitive and Affective Neuroscience, 9*(10), 1458–1463. 10.1093/scan/nst132. 10.1093/scan/nst132 PMC4187259 23946004

[CR41] Jones, D. T., Knopman, D. S., Gunter, J. L., Graff-Radford, J., Vemuri, P., Boeve, B. F., … Alzheimer’s Disease Neuroimaging Initiative. (2016). Cascading network failure across the Alzheimer's disease spectrum. *Brain, 139*(Pt 2), 547–562. 10.1093/brain/awv338 10.1093/brain/awv338 PMC4805086 26586695

[CR42] Jorm AF (2004). The informant questionnaire on cognitive decline in the elderly (IQCODE): A review. International Psychogeriatrics.

[CR43] Klaassens BL, van Gerven JMA, van der Grond J, de Vos F, Möller C, Rombouts SARB (2017). Diminished posterior Precuneus connectivity with the default mode network differentiates Normal aging from Alzheimer's disease. Frontiers in Aging Neuroscience.

[CR44] Langer KG, Levine DN (2014). Babinski, J. (1914). Contribution to the study of the mental disorders in hemiplegia of organic cerebral origin (Anosognosia). Translated by K.G. Langer & D.N. Levine translated from the original contribution à l'Étude des troubles Mentaux dans l'Hémiplégie Organique Cérébrale (Anosognosie). Cortex.

[CR45] Lau WK, Leung MK, Lee TM, Law AC (2016). Resting-state abnormalities in amnestic mild cognitive impairment: A meta-analysis. Translational Psychiatry.

[CR46] Leech R, Sharp DJ (2014). The role of the posterior cingulate cortex in cognition and disease. Brain.

[CR47] Mak E, Chin R, Ng LT, Yeo D, Hameed S (2015). Clinical associations of anosognosia in mild cognitive impairment and Alzheimer's disease. International Journal of Geriatric Psychiatry.

[CR48] Marková IS, Berrios GE (2014). The construction of anosognosia: History and implications. Cortex.

[CR49] Markowitsch HJ, Staniloiu A (2011). Memory, autonoetic consciousness, and the self. Conscioussness and cognition.

[CR50] McKhann G, Drachman D, Folstein M, Katzman R, Price D, Stadlan EM (1984). Clinical diagnosis of Alzheimer’s disease: Report of the NINCDS-ADRDA work group under the auspices of Department of Health and Human Services Task Force on Alzheimer’s disease. Neurology.

[CR51] McKhann GM, Knopman DS, Chertkow H, Hyman BT, Jack CR, Kawas CH (2011). The diagnosis of dementia due to Alzheimer's disease: Recommendations from the National Institute on Aging-Alzheimer's Association workgroups on diagnostic guidelines for Alzheimer's disease. Alzheimer's & Dementia.

[CR52] Migliorelli R, Teson A, Sabe L, Petracca G, Petracchi M, Leiguarda R, Starkstein SE (1995). Anosognosia in Alzheimer’s disease: A study of associated factors. The Journal of Neuropsychiatry and Clinical Neuroscience.

[CR53] Mimura M, Yano M (2006). Memory impairment and awareness of memory deficits in early-stage Alzheimer's disease. Reviews in the Neurosciences.

[CR54] Mograbi DC, Brown RG, Morris RG (2009). Anosognosia in Alzheimer's disease--the petrified self. Consciousness and Cognition.

[CR55] Mograbi DC, Ferri CP, Sosa AL, Stewart R, Laks J, Brown R, Morris RG (2012). Unawareness of memory impairment in dementia: A population-based study. International Psychogeriatrics.

[CR56] Moher D, Liberati A, Tetzlaff J, Altman DG, PRISMA Group (2009). Preferred reporting items for systematic reviews and meta-analyses: The PRISMA statement. Annals of Internal Medicine.

[CR57] Morris RG, Mograbi DC (2013). Anosognosia, autobiographical memory and self knowledge in Alzheimer's disease. Cortex.

[CR58] Nobili, F., Mazzei, D., Dessi, B., Morbelli, S., Brugnolo, A., Barbieri, P., … Pagani, M. (2010). Unawareness of memory deficit in amnestic MCI: FDG-PET findings. *Journal of Alzheimer's Disease, 22*(3), 993–1003. 10.3233/JAD-2010-100423 10.3233/JAD-2010-100423 20858977

[CR59] Northoff G, Heinzel A, de Greck M, Bermpohl F, Dobrowolny H, Panksepp J (2006). Self-referential processing in our brain--a meta-analysis of imaging studies on the self. Neuroimage.

[CR60] Northoff G, Qin P, Feinberg TE (2011). Brain imaging of the self- conceptual, anatomical and methodological issues. Consciousness and Cognition.

[CR61] Nurmi Laihosalo ME, Jehkonen M (2014). Assessing anosognosias after stroke: A review of the methods used and developed over the past 35 years. Cortex.

[CR62] Oba, H., Matsuoka, T., Imai, A., Fujimoto, H., Kato, Y., Shibata, K., Nakamura K., Narumoto, J. (2018). Interaction between memory impairment and depressive symptoms can exacerbate anosognosia: A comparison of Alzheimer's disease with mild cognitive impairment. *Aging & Mental Health*, 1–7. 10.1080/13607863.2018.1442411. 10.1080/13607863.2018.1442411 29528693

[CR63] Orfei MD, Piras F, Macci E, Caltagirone C, Spalletta G (2013). The neuroanatomical correlates of cognitive insight in schizophrenia. Social Cognitive and Affective Neuroscience.

[CR64] Orfei MD, Robinson RG, Bria P, Caltagirone C, Spalletta G (2008). Unawareness of illness in neuropsychiatric disorders: Phenomenological certainty versus etiopathogenic vagueness. Neuroscientist.

[CR65] Orfei MD, Varsi AE, Blundo C, Celia E, Casini AR, Caltagirone C, Spalletta G (2010). Anosognosia in mild cognitive impairment and mild Alzheimer's disease: Frequency and neuropsychological correlates. The American Journal of Geriatric Psychiatry.

[CR66] Ott BR, Noto RB, Fogel BS (1996). Apathy and loss of insight in Alzheimer's disease: A SPECT imaging study. The Journal of Neuropsychiatry and Clinical Neuroscience.

[CR67] Perrotin, A., Desgranges, B., Landeau, B., Mézenge, F., La Joie, R., Egret, S., … Chételat, G. (2015). Anosognosia in Alzheimer disease: Disconnection between memory and self-related brain networks. *Annals of Neurology, 78*(3), 477–486. 10.1002/ana.24462. 10.1002/ana.24462 26085009

[CR68] Petersen, R. C., Doody, R., Kurz, A., Mohs, R. C., Morris, J. C., Rabins, P. V., … B. (2001). Current concepts in mild cognitive impairment. *Archives of Neurology, 58*(12), 1985–1992. 10.1001/archneur.58.12.1985 10.1001/archneur.58.12.1985 11735772

[CR69] Piras F, Piras F, Orfei MD, Caltagirone C, Spalletta G (2016). Self-awareness in mild cognitive impairment: Quantitative evidence from systematic review and meta-analysis. Neuroscience and Biobehavioral Reviews.

[CR70] Prigatano GP (2014). Anosognosia and patterns of impaired self-awareness observed in clinical practice. Cortex.

[CR71] Reed BR, Jagust WJ, Coulter L (1993). Anosognosia in Alzheimer's disease: Relationships to depression, cognitive function, and cerebral perfusion. Journal of Clinical and Experimental Neuropsychology.

[CR72] Reitsma, J.B., Rutjes, A.W.S., Whiting, P., Vlassov, V.V., Leeflang, M.M.G., & Deeks, J.J. (2009). Chapter 9: Assessing methodological quality. In: Deeks, J.J., Bossuyt, P.M., & Gatsonis, C. (eds.), *Cochrane handbook for systematic reviews of diagnostic test accuracy version 1.0.0*. The Cochrane collaboration, Available from: http://srdta.cochrane.org/

[CR73] Ries, M. L., Jabbar, B. M., Schmitz, T. W., Trivedi, M. A., Gleason, C. E., Carlsson, C. M., … Johnson S. C. (2007). Anosognosia in mild cognitive impairment: Relationship to activation of cortical midline structures involved in self-appraisal. *Journal of the International Neuropsychological Society, 13*(3), 450–461. 10.1017/S1355617707070488. 10.1017/S1355617707070488 PMC2654607 17445294

[CR74] Ries, M. L., McLaren, D. G., Bendlin, B. B., Guofanxu, Rowley, H. A., Birn, R. … Johnson, S.C. (2012). Medial prefrontal functional connectivity--relation to memory self-appraisal accuracy in older adults with and without memory disorders. *Neuropsychologia, 50*(5), 603–611. 10.1016/j.neuropsychologia.2011.12.014. 10.1016/j.neuropsychologia.2011.12.014 PMC3537182 22230228

[CR75] Ries ML, Schmitz TW, Kawahara TN, Torgerson BM, Trivedi MA, Johnson SC (2006). Task-dependent posterior cingulate activation in mild cognitive impairment. Neuroimage.

[CR76] Ruby P, Collette F, D'Argembeau A, Péters F, Degueldre C, Balteau E (2009). Perspective taking to assess self-personality: what's modified in Alzheimer's disease?. Neurobiology of Aging.

[CR77] Salmon, E., Perani, D., Herholz, K., Marique, P., Kalbe, E., Holthoff, V., … Garraux, G. (2006). Neural correlates of anosognosia for cognitive impairment in Alzheimer's disease. *Human Brain Mapping, 27*(7), 588–597. 10.1002/hbm.20203 10.1002/hbm.20203 PMC6871369 16247783

[CR78] Sedaghat F, Dedousi E, Baloyannis I, Tegos T, Costa V, Dimitriadis AS, Baloyannis SJ (2010). Brain SPECT findings of anosognosia in Alzheimer's disease. Journal of Alzheimer's Disease.

[CR79] Shibata K, Narumoto J, Kitabayashi Y, Ushijima Y, Fukui K (2008). Correlation between anosognosia and regional cerebral blood flow in Alzheimer's disease. Neuroscience Letters.

[CR80] Spalletta G, Girardi P, Caltagirone C, Orfei MD (2012). Anosognosia and neuropsychiatric symptoms and disorders in mild Alzheimer disease and mild cognitive impairment. Journal of Alzheimer's Disease.

[CR81] Spalletta, G., Piras, F., Piras, F., Sancesario, G., Iorio, M., Fratangeli, C., … Orfei, M. D. (2014). Neuroanatomical correlates of awareness of illness in patients with amnestic mild cognitive impairment who will or will not convert to Alzheimer's disease. *Cortex, 61*, 183–195. 10.1016/j.cortex.2014.10.010 10.1016/j.cortex.2014.10.010 25481475

[CR82] Sperling, R. A., Aisen, P. S., Beckett, L. A., Bennett, D. A., Craft, S., Fagan, A. M., … Phelps, C. H. (2011). Toward defining the preclinical stages of Alzheimer's disease: Recommendations from the National Institute on Aging-Alzheimer's Association workgroups on diagnostic guidelines for Alzheimer's disease. *Alzheimer's & Dementia, 7*(3), 280–292. 10.1016/j.jalz.2011.03.003 10.1016/j.jalz.2011.03.003 PMC3220946 21514248

[CR83] Starkstein, S .E., Sabe, L., Vazquez, S., Teson, A., Petracca, G., Chemerinski, E., … Leiguarda, R. (1996). Neuropsychological, psychiatric, and cerebral blood flow findings in vascular dementia and Alzheimer's disease. *Stroke, 27*(3), 408–414. 10.1161/01.STR.27.3.408. 10.1161/01.str.27.3.408 8610304

[CR84] Starkstein SE (2014). Anosognosia in Alzheimer's disease: Diagnosis, frequency, mechanism and clinical correlates. Cortex.

[CR85] Starkstein SE, Brockman S, Bruce D, Petracca G (2010). Anosognosia is a significant predictor of apathy in Alzheimer's disease. The Journal of Neuropsychiatry and Clinical Neuroscience.

[CR86] Starkstein SE, Vázquez S, Migliorelli R, Tesón A, Sabe L, Leiguarda R (1995). A single-photon emission computed tomographic study of anosognosia in Alzheimer's disease. Archives of Neurology.

[CR87] Sultzer DL, Leskin LP, Melrose RJ, Harwood DG, Narvaez TA, Ando TK, Mandelkern MA (2014). Neurobiology of delusions, memory, and insight in Alzheimer disease. The American Journal of Geriatric Psychiatry.

[CR88] Symons CS, Johnson BT (1997). The self-reference effect in memory: A meta-analysis. Psychological Bulletin.

[CR89] Tagai K, Shinagawa S, Kada H, Inamura K, Nagata T, Nakayama K (2018). Anosognosia in mild Alzheimer's disease is correlated with not only neural dysfunction but also compensation. Psychogeriatrics.

[CR90] The Nordic Cochrane Centre (2019). Review manager (RevMan) [computer program]. (2008) version 5.0.

[CR91] Therriault, J., Ng, K. P., Pascoal, T. A., Mathotaarachchi, S., Kang, M. S., Struyfs, H., … Alzheimer's Disease Neuroimaging Initiative. (2018). Anosognosia predicts default mode network hypometabolism and clinical progression to dementia. *Neurology, 90*(11), e932–e939. 10.1212/WNL.0000000000005120 10.1212/WNL.0000000000005120 PMC5858945 29444971

[CR92] Turnbull OH, Fotopoulou A, Solms M (2014). Anosognosia as motivated unawareness: The 'defence' hypothesis revisited. Cortex.

[CR93] Turró-Garriga O, Garre-Olmo J, Calvó-Perxas L, Reñé-Ramírez R, Gascón-Bayarri J, Conde-Sala JL (2016). Course and determinants of Anosognosia in Alzheimer's disease: A 12-month follow-up. Journal of Alzheimer's Disease.

[CR94] Vallet GT, Hudon C, Simard M, Versace R (2013). The disconnection syndrome in the Alzheimer's disease: The cross-modal priming example. Cortex.

[CR95] Vandekerckhove M, Panksepp J (2011). A neurocognitive theory of higher mental emergence: From anoetic effective experiences to noetic knowledge and autonoetic awareness. Neuroscience and Biobehavioral Reviews.

[CR96] Vannini, P., Hanseeuw, B., Munro, C. E., Amariglio, R. E., Marshall, G. A., Rentz, D. M., … Sperling, R. A. (2017). Anosognosia for memory deficits in mild cognitive impairment: Insight into the neural mechanism using functional and molecular imaging. *Neuroimage. Clinical, 15*, 408–414. 10.1016/j.nicl.2017.05.020 10.1016/j.nicl.2017.05.020 PMC5458095 28616381

[CR97] Vogel A, Hasselbalch SG, Gade A, Ziebell M, Waldemar G (2005). Cognitive and functional neuroimaging correlate for anosognosia in mild cognitive impairment and Alzheimer's disease. International Journal of Geriatric Psychiatry.

[CR98] Vuilleumier P (2004). Anosognosia: The neurology of beliefs and uncertainties. Cortex.

[CR99] Wang, J., Zuo, X., Dai, Z., Xia, M., Zhao, Z., Zhao, X., … Y. (2013). Disrupted functional brain connectome in individuals at risk for Alzheimer's disease. *Biological Psychiatry, 73*(5), 472–481. 10.1016/j.biopsych.2012.03.026 10.1016/j.biopsych.2012.03.026 22537793

[CR100] Wang, L., Li, H., Liang, Y., Zhang, J., Li, X., Shu, N., … Zhang, Z. (2013). Amnestic mild cognitive impairment: Topological reorganization of the default-mode network. *Radiology, 268*(2), 501–514. 10.1148/radiol.13121573 10.1148/radiol.13121573 23481166

[CR101] Weiler M, Northoff G, Damasceno BP, Balthazar ML (2016). Self, cortical midline structures and the resting state: Implications for Alzheimer's disease. Neuroscience and Biobehavioral Reviews.

[CR102] Whitfield-Gabrieli S, Moran JM, Nieto-Castañón A, Triantafyllou C, Saxe R, Gabrieli JD (2011). Associations and dissociations between default and self-reference networks in the human brain. Neuroimage.

[CR103] Wilson B, Cockburn J, Baddeley A, Hiorns R (1989). The development and validation of a test battery for detecting and monitoring everyday memory problems. Journal of Clinical and Experimental Neuropsychology.

[CR104] Wilson RS, Sytsma J, Barnes LL, Boyle PA (2016). Anosognosia in Dementia. Current Neurology and Neuroscience Reports.

[CR105] Winblad, B., Palmer, K., Kivipelto, M., Jelic, V., Fratiglioni, L., Wahlund, L. O., … R.C. (2004). Mild cognitive impairment--beyond controversies, towards a consensus: Report of the international working group on mild cognitive impairment. *Journal of Internal Medicine, 256*(3), 240–246. 10.1111/j.1365-2796.2004.01380.x 10.1111/j.1365-2796.2004.01380.x 15324367

[CR106] Yoon, B., Shim, Y. S., Hong, Y. J., Choi, S. H., Park, H. K, Park, S. A., … Yang, D.W. (2017). Anosognosia and its relation to psychiatric symptoms in early-onset Alzheimer disease. *Journal of Geriatric Psychiatry and Neurology, 30*(3), 170–177. 10.1177/0891988717700508. 10.1177/0891988717700508 28421896

[CR107] Zamboni, G., Drazich, E., McCulloch, E., Filippini, N., Mackay, C. E., Jenkinson, M., … Wilcock, G. K. (2013). Neuroanatomy of impaired self-awareness in Alzheimer's disease and mild cognitive impairment. *Cortex, 49*(3), 668–678. 10.1016/j.cortex.2012.04.011 10.1016/j.cortex.2012.04.011 22677047

[CR108] Zamboni G, Wilcock G (2011). Lack of awareness of symptoms in people with dementia: The structural and functional basis. International Journal of Geriatric Psychiatry.

[CR109] Zhu, H., Zhou, P., Alcauter, S., Chen, Y., Cao, H., Tian, M., … W. (2016). Changes of intranetwork and internetwork functional connectivity in Alzheimer's disease and mild cognitive impairment. *Journal of Neural Engineering, 13*(4), 046008. 10.1088/1741-2560/13/4/046008 10.1088/1741-2560/13/4/046008 27247279

