# Supplementary material for: Functional Neural Correlates of Anosognosia in Mild Cognitive Impairment and Alzheimer’s Disease: a Systematic Review
Source: Neuropsychol Rev. 2019 Jun 3;29(2):139–65. doi: 10.1007/s11065-019-09410-x (PMC6560017; doi:10.1007/s11065-019-09410-x)
Supplement: Supplementary file 1 — (DOCX 310 kb) [file 11065_2019_9410_MOESM1_ESM.docx]

1. **Risk of bias assessment**

**Risk of bias in brain perfusion studies**

Among the brain perfusion studies, the risk of bias for patient selection is present in only one article (Mimura and Yano, 2006) and unclear in another (Starkstein et al., 1995). Mimura and Yano include two different control groups, one for the imaging analysis and the other for the recognition/awareness examination; this design increases the risk of diagnostic case-control bias as different selection procedures are used to sample patients with anosognosia and control groups. The risk of recovery bias is also present in this study since 75% of AD patients were taking donepezil (Mimura and Yano, 2006). The conduct of the study or interpretation of the SPECT results had an unclear risk of bias in one study since it was not specified whether the rater was blinded to the patient’s anosognosia classification nor was a threshold for the PET and SPECT standardized uptake value specified (Sedaghat et al., 2010). All brain perfusion articles have a low risk of introducing bias due to the interpretation or execution of the anosognosia screening instrument. The risk of bias is low because the anosognosia screening instruments adequately assess the target condition; concomitantly the interpretation of the results was performed without knowledge of the neuroimaging results. One article addressed the risk for verification bias by having the informants undergo a thorough neuropsychological evaluation (Tagai et al., 2018). The patient flow and timing has a high risk of introducing bias in two studies (Starkstein et al., 1995; Vogel et al., 2005), while three articles have an unclear risk (Reed et al., 1993; Sedaghat et al., 2010; Shibata et al., 2008). In the first study, patients with an intermediate AQ-D score (15-31) were excluded from the neuroimaging analysis; leading to high risk of an uncertain results bias (Starkstein et al., 1995). In the second study, uncertain results bias is possible as 11 eligible patients are not included in the neuroimaging analysis; nonetheless, the probability is minor as the patients that underwent the SPECT study were randomly selected (Vogel et al., 2005). The article also has a risk of introducing disease progression bias, as there is an unspecified elapsed time between neuropsychological and neuroimaging assessment (Vogel et al., 2005). The risk of disease progression bias is also present in the three articles with an unclear risk of bias because the time interval between neuropsychological assessment and neuroimaging was not specified (Reed et al., 1993; Sedaghat et al., 2010; Shibata et al., 2008).

**Risk of bias in brain metabolism studies**

None of the brain metabolism studies had increased patient selection risk of bias as they performed purposive sampling; all avoided case-control bias, avoided inappropriate exclusions and the patient characteristics matched the review question. After contact with the authors, the conduct or interpretation of the FDG-PET neuroimaging technique does not introduce a high risk of bias as the raters were blinded to the patient’s anosognosia classification and imaging thresholds for neuroimaging classification were specified in other articles from that group (Jedidi et al., 2014). Two articles used the Everyday Cognition Scale to assess anosognosia, which is not a standard practice (Gerretsen et al., 2017; Therriault et al., 2018). All brain metabolism articles have a low risk of introducing bias associated with the interpretation or conduct of the anosognosia screening instrument, as well as a low risk associated with patient flow and timing.

**Risk of bias in brain activation studies**

Patient selection risk of bias is low in all included brain activation studies. In two articles (Amanzio et al., 2011; Zamboni et al., 2013), the conduct or interpretation of the fMRI activation studies introduces an unclear risk of bias as it is not indicated if the imaging rater is blinded to the patient’s anosognosia classification nor was a threshold for neuroimaging classification specified as in other functional neuroimaging techniques. All brain activation articles have a low risk of introducing bias associated with the interpretation or conduct of the anosognosia screening instrument. All articles have a low risk of disease progression bias since the neuropsychological evaluation and neuroimaging studies were performed within days.

**Risk of bias in brain connectivity studies**

None of the brain connectivity studies has increased patient selection risk of bias. The conduct or interpretation of the four fMRI connectivity neuroimaging studies introduces a low risk of bias as imaging thresholds are specified in the connectivity analysis. All brain connectivity studies have a low risk of introducing bias associated with the interpretation or conduct of the anosognosia screening instrument. Patient flow and timing risk of bias are present in one article (Berlingeri et al., 2015). The risk of bias due to withdrawal is present as two AD patients with anosognosia, four AD patients without anosognosia and five healthy controls withdrew from the neuroimaging portion of the study; affecting test performance since withdrawals accounted for a quarter of the subjects (Berlingeri et al., 2015). In this same article, disease progression bias cannot be discarded as the time between neuropsychological and neuroimaging assessment is not specified (Berlingeri et al., 2015).

1. **Risk of bias analysis**

The risk of bias assessment for this systematic review can be grouped into four domains, patient selection, neuroimaging technique, anosognosia screening instrument, and flow and timing. There was no need to exclude studies from our analysis based on the risk of bias, as no study had high risk of bias in more than one domain. Overall, the patient spectrum of the included studies in this review included memory clinic outpatients, which is considered an adequate source for purposive sampling in dementia studies. Yet, selection bias might be present and prevent the generalization of the findings to community-based patients. Only one article had high risk of bias for its patient selection process since two different control groups, one for the imaging and the other for the recognition/awareness examination, were used; this design increases the risk of diagnostic case-control bias as different selection processes were used to sample patients with anosognosia and control groups (Mimura and Yano, 2006). The risk of bias regarding the applicability of the neuroimaging technique was low or unclear in all studies. For the studies that had unclear risk this was due to the omission of methodological detail, such as blinding of the imaging rater or adequate implementation of imaging thresholds from previously established data (Amanzio et al., 2011; Sedaghat et al., 2010; Zamboni et al., 2013). Regarding correctly targeted anosognosia screening instruments, verification bias cannot be excluded in all studies. Methods to assess anosognosia that use a participant-informant discrepancy score, and semi-structured interviews to a certain extent, are subjective measures of anosognosia. Informants may not be completely unbiased when providing an estimate of the patient’s awareness of memory deficit, as self-report scales can potentially be a skewed product of the informant’s over- or underestimation of the patient’s abilities (Trosset and Kaszniak, 1996). Objective metamemory testing, like the use of the recognition-based episodic memory-monitoring task, is preferable to subjective assessment of the self (Cosentino et al., 2007). Four of the reviewed articles based their assessment of anosognosia through a judgment of test performance task to measure self-accuracy discrepancy; however, eighteen studies used a discrepancy score between the patient and an informant and four assessed anosognosia through a semi-structured interview, which depends on the patient’s and caregivers’ subjectivity and hence introduces verification bias. In addition, two articles used an instrument (i.e. Everyday Cognition Scale) that was not designed to assess self-awareness (Gerretsen et al., 2017; Therriault et al., 2018). To reduce verification bias, three studies applied the Zarit's burden interview to control for personal strain and role strain of the caregiver (Jedidi et al., 2014; Ries et al., 2012; Tagai et al., 2018), while one study performed a full neuropsychological evaluation on the informants (Amanzio et al., 2011). Flow and timing regarding the awareness assessment and functional neuroimaging acquisition particularly take into consideration disease progression when dealing with memory decline. Among the reviewed studies, none had a high risk of bias but a quarter had an unclear risk due to the time interval between anosognosia evaluation, reporting omission and the neuroimaging test. Regularly studies undergo population attrition due to patient withdrawal affecting test performance (Reitsma et al., 2009). In one study this could have been an issue, as the withdrawal of two AD patients with anosognosia, four AD patients without anosognosia and five healthy controls accounted for a quarter of the population (Berlingeri et al., 2015). Future studies attempting to reduce the risk of bias in the patient selection process for the study of anosognosia or unawareness of memory deficits need to consider the population, the setting, and the recruitment strategy to obtain generalizable findings. At the same time, blinding is necessary during the evaluation of anosognosia and the neuroimaging assessment. Generalizability of the results might be accomplished by reducing the between-study variability, which can be accomplished by setting imaging thresholds; following standardized imaging protocols and utilizing validated and standardized awareness screening instruments. Meanwhile, the flow and timing in the studies should always take into consideration an acceptable time window for clinical, neuropsychological and imaging evaluation. Assessment of the risk of bias for all brain perfusion, metabolism activation, and connectivity studies can be found in Supplemental Table 1 and Supplemental figures 1 and 2.

1. **Specific limitations**

Differences in the type of information used in diagnosing anosognosia exist due to the variability of sources; namely, the patient, the caregiver or the clinician (Nurmi and Jehkonen, 2014). Three methods are primarily employed to assess anosognosia: 1) measurement instruments that incorporate a discrepancy score between patient and an informant; 2) measurement instruments based on a self-accuracy discrepancy score, in which the patient prospectively attempts to predict their performance on a neuropsychological test; and 3) measurement based on the examiner’s judgment. Overestimation of subjective memory complaints and underestimation of memory impairment are a contributing factor to the variability of anosognosia diagnosis in AD patients (Morris and Mograbi, 2013). In this respect, instruments that objectively measure awareness of memory deficit are of interest. While subjective rating discrepancy (SRD) employs parallel informant rating discrepancy scores to quantify the extent of anosognosia, objective-judgment discrepancy (OJD) assesses anosognosia by measuring the difference between performance and judgment (Hannesdottir and Morris, 2007). It is hypothesized that SRD utilizes accumulated knowledge and requires integration of information over an extended period; whereas, OJD assesses a more immediate type of awareness which is based on the ability to evaluate ongoing memory performance (Hannesdottir and Morris, 2007). Although SRD Global rating scale correlates with SRD Memory scale, experimenter rating scales (ERS) only correlate with SRD Global rating scale and not to the SRD Memory scale; therefore, suggesting that the ERS and the SRD Global rating scale provide an overview of anosognosia, while the SRD Memory scale explores specifically the memory domain (Hannesdottir and Morris, 2007). These instruments measure different domains of anosognosia, which limits the comparability of the results; thus, possibly leading to an over-interpretation of the results.

The use of anti-cholinesterase inhibitors (AChEIs) could be a confounding variable in some of the studies in this review. In a pilot study using an event-related delayed-response visual memory task for novel faces, donepezil increased activation in the ventrolateral prefrontal cortex versus placebo in MCI patients (Petrella et al., 2009). Increased connectivity among AD patients undergoing treatment with donepezil and memantine has been reported. While use of memantine was associated with greater connectivity between DMN and precuneus, the use of donepezil was associated with increased connectivity between the hippocampal network and PCC, middle and inferior frontal gyri and dorsolateral PFC, the midcingulate cortex network and ventral mPFC, precuneus, ventral ACC, precentral gyrus, inferior parietal cortex and parahippocampus, and the PCC network and ventral ACC and cuneus (Dennis and Thompson, 2014). In the present review, six studies included and reported patients taking AChEIs, seven did not report if included patients were taking AChEIs, while three articles reported that none of the subjects were taking AChEIs. The number of patients taking AChEIs, the population studied and the functional neuroimaging technique varied in these studies. In two SPECT studies, Vogel and colleagues (2005) included 18 patients from a cohort of 45 AD/aMCI and Hanyu and colleagues (2008) included five unaware and seven aware AD patients who were taking donepezil. Among the PET studies, Salmon and colleagues (2006) included 89 out of 209 mild to moderate AD patients who were taking AChEIs and Sultzer and colleagues (2014) included 32 out of 80 AD patients who were taking AChEIs. In two fMRI activation studies, all 16 MCI patients included were taking AChEIs (Rise et al., 2007) and all 14 AD patients were taking AChEIs (Ruby et al., 2009). Even though Petrella and colleagues (2009) report cortical activation differences in MCI patients taking donepezil, many clinical trials testing the efficacy of donepezil, galantamine, rivastigmine, and rofecoxib have failed to show efficacy preventing worsening of performance over time in MCI patients; tracked on the Alzheimer’s Disease Assessment Scale-cognitive subscale, MMSE, and Clinical Dementia Rating-sum of boxes (Petersen et al., 2017). The inclusion of subject taking AChEIs limits the reliability of six studies as the neuroimaging outcome measure of the presented results might be unreliable; as well as the accuracy of clinical staging might be compromised.

In this review, four SPECT studies used 99mTc-HMPAO, four 123I-IMP and one 99mTc-ECD. Although the three radioligands are all suitable for detecting perfusion differences between AD and control groups, the perfusion topography might vary due to the different brain uptake mechanisms of each radioligand. Due to differences in their biochemical properties, SPECT radioligands yield different brain uptake patterns. In AD patients, differences in perfusion areas have been reported between 99mTc-HMPAO and 99mTc-ECD; albeit not hindering correct classification of patients (van Dyck et al., 1996). Among those regions are the right and left occipital cuneus, the left occipital and parietal precuneus, the left superior and middle temporal gyri, and the hippocampi in both hemispheres (Koulibaly et al., 2003). Brain uptake differences among these two radioligands have also been reported in AD patients undergoing a verbal memory task (Nobili et al., 2007). To our knowledge only one report compares 123I-IMP brain uptake to 99mTc-HMPAO, reporting that either radioligand can be used for the differential diagnosis of dementia (Gemmell et al., 1988).

**References**

Cosentino S, Metcalfe J, Butterfield B, Stern Y. (2007). Objective metamemory testing captures awareness of

deficit in Alzheimer's disease. Cortex; 43(7): 1004-19. doi: 10.1016/S0010-9452(08)70697-X

Gemmell HG, Sharp PF, Besson JA, et al. (1988). A comparison of Tc-99m HM-PAO and I-123 IMP cerebral

SPECT images in Alzheimer's disease and multi-infarct dementia. Eur J Nucl Med.; 14(9-10): 463-6. doi: 10.1007/BF00252389.

Hannesdottir K, Morris RG. (2007). Primary and secondary anosognosia for memory impairment in patients with

Alzheimer's disease. Cortex; 43(7): 1020-30. doi: 10.1016/S0010-9452(08)70698-1.

Koulibaly PM, Nobili F, Migneco O, et al. (2003). 99mTc-HMPAO and 99mTc-ECD perform differently in

typically hypoperfused areas in Alzheimer's disease. Eur J Nucl Med Mol Imaging; 30(7): 1009-13. Epub 2003 May 15. doi: 10.1007/s00259-003-1193-2.

Nobili F, Koulibaly PM, Rodriguez G, et al. (2007). 99mTc-HMPAO and 99mTc-ECD brain uptake correlates

of verbal memory in Alzheimer's disease. Q J Nucl Med Mol Imaging.; 51(4): 357-63.

Petersen RC, Thomas RG, Aisen PS, et al. (2017). Randomized controlled trials in mild cognitive impairment:

Sources of variability. Neurology; 88(18): 1751-58. doi: 10.1212/WNL.0000000000003907. Epub 2017 Apr 5.

Petrella JR, Prince SE, Krishnan S, et al. (2009). Effects of donepezil on cortical activation in mild cognitive

impairment: a pilot double-blind placebo-controlled trial using functional MR imaging. AJNR Am J Neuroradiol.; 30(2): 411-6. doi: 10.3174/ajnr.A1359. Epub 2008 Nov 11.

Trosset MW, Kaszniak AW. (1996). Measures of deficit unawareness for predicted performance experiments. J

Int Neuropsychol Soc.; 2(4): 315-22. doi: 10.1017/S1355617700001338.

van Dyck CH, Lin CH, Smith EO, et al. (1996). Comparison of technetium-99m-HMPAO and technetium-99m-

ECD cerebral SPECT images in Alzheimer's disease. J Nucl Med.; 37(11): 1749-55. ISSN: 0161-5505

**Supplemental Table 1. Risk of bias assessment table**

| **Reference** | **Patient selection** | **Neuroimaging technique** | | | **Anosognosia screening instrument** | **Flow and timing** |
| --- | --- | --- | --- | --- | --- | --- |
| **SPECT** | | | | | | |
| Reed et al., 1993 | Low risk | | Low risk | Low risk | | Unclear risk |
| Starkstein et al., 1995 | Unclear risk | | Low risk | Low risk | | Unclear risk |
| Starkstein et al., 1996 | Low risk | | Low risk | Low risk | | Low risk |
| Derouesné et al., 1999 | Low risk | | Low risk | Low risk | | Low risk |
| Vogel et al., 2005 | Low risk | | Low risk | Low risk | | Unclear risk |
| Mimura et al., 2006 | High risk | | Low risk | Low risk | | Low risk |
| Hanyu et al., 2008 | Low risk | | Low risk | Low risk | | Low risk |
| Shibata et al., 2008 | Low risk | | Low risk | Low risk | | Low risk |
| Sedaghat et al., 2010 | Low risk | | Unclear risk | Low risk | | Low risk |
| Tagai et al., 2018 | Low risk | | Low risk | Low risk | | Low risk |
| **PET** | | | | | | |
| Harwood et al., 2005 | Low risk | | Low risk | Low risk | | Low risk |
| Salmon et al., 2006 | Low risk | | Low risk | Low risk | | Low risk |
| Nobili et al., 2010 | Low risk | | Low risk | Low risk | | Low risk |
| Jedidi et al., 2014 | Low risk | | Low risk | Low risk | | Low risk |
| Sultzer et al., 2014 | Low risk | | Low risk | Low risk | | Low risk |
| Gerretsen et al., 2017 | Low risk | | Low risk | Low risk | | Low risk |
| Therriault et al., 2018 | Low risk | | Low risk | Low risk | | Low risk |
| **fMRI activation** | | | | | | |
| Ries et al., 2007 | Low risk | | Low risk | | Low risk | Low risk |
| Ruby et al., 2009 | Low risk | | Low risk | | Low risk | Low risk |
| Amanzio et al., 2011 | Low risk | | Unclear risk | | Low risk | Low risk |
| Zamboni et al., 2013 | Low risk | | Unclear risk | | Low risk | Low risk |
| **fMRI connectivity** | | | | | | |
| Ries et al., 2012 | Low risk | | Low risk | | Low risk | Low risk |
| Berlingeri et al., 2015 | Low risk | | Low risk | | Low risk | Unclear risk |
| Perrotin et al., 2015 | Low risk | | Low risk | | Low risk | Low risk |
| Vannini et al., 2017 | Low risk | | Low risk | | Low risk | Low risk |

**Supplemental Table 2.** Additional characteristics of perfusion studies

| **Reference** | **Cognition screening instrument** | **Diagnosis criteria** | **Anosognosia measurement instrument** | **Method used to assess awareness** | **Note** |
| --- | --- | --- | --- | --- | --- |
| Reed et al., 1993 | MMSE | NINCDS-ADRDA | Anosognosia clinical rating scale | Consensus clinical rating for degree of awareness of memory loss | Patients taking antidepressants or antipsychotics excluded; depression sub-analysis. Dose not screen for vascular disease burden |
| Starkstein et al., 1995 | MMSE | NINCDS-ADRDA | AQ-D | Discrepancy score between patient and informant | Patients underwent Hamilton depression scale and functional independence scale. Hachinski score <4. |
| Starkstein et al., 1996 | MMSE | NINCDS-ADRDA | AQ-D | Discrepancy score between patient and informant | Hachinski score: 6.8 (5.7) in IVD; 1.3 (2.0) in AD. AQ-D score: 24.2 (23.6) in IVD; 12.8 (17.2) in AD, underwent MRI and CT as well. |
| Derouesné et al., 1999 | MMSE | NINCDS-ADRDA | Cognitive Difficulties Scale and Anosognosia clinical rating scale | Discrepancy score between patient and informant | MMSE ≥ 18 |
| Vogel et al., 2005 | MMSE and CDR | NINCDS-ADRDA and Petersen et al., 2001 | Memory Questionnaire | Discrepancy score between patient and informant | aMCI: MMSE >20, CDR= 0.5. aMCI with CDR 0.5: 30. AD with CDR 0.5: 21; CDR 1: 13; CDR 2: 1; CDR 3: 1 |
| Mimura et al., 2006 | MMSE and CDR | NINCDS-ADRDA and DSM-IV | Awareness of memory experimental paradigm | Self-accuracy discrepancy | CDR 1: 19, CDR 2: 5. 18 taking donepezil, none taking antidepressant, anxiolytics or antipsychotics. SPECT control group different that behavioral control group |
| Hanyu et al., 2008 | MMSE and CDR | NINCDS-ADRDA | Every day Memory Checklist | Discrepancy score between patient and informant | All AD patients with MMSE >24 and CDR=0.5; Five unaware and 7 aware patients taking donepezil. |
| Shibata et al., 2008 | MMSE | NINCDS-ADRDA | Squire and Zouzounis Questionnaire | Discrepancy score between patient and informant | No patients on AChEIs or psychotropic drugs. MMSE>17 |
| Sedaghat et al., 2010 | MMSE and CDR | NINCDS-ADRDA | Non-structured interview | Non-structured interview | Hachinski score<7; MMSE 20-26 considered mild, 10-19 considered moderate |
| Tagai et al., 2018 | MMSE and CDR | NIA-AA | AQ-D | Discrepancy score between patient and informant | Zarit's burden interview to the caregivers. All participants with CDR ≤1. |

Abbreviations: AChEIs: acetylcholinesterase inhibitors; AD: Alzheimer’s Disease; aMCI: amnestic mild cognitive impairment; AQ-D: Anosognosia Questionnaire Dementia; CDR: Clinical Dementia Rating; CT: computed tomography; DSM-IV: Diagnostic and Statistical Manual of Mental Disorders- IV; IVD: ischemic vascular dementia; MMSE: Mini-mental state examination; MRI: magnetic resonance imaging; NIA-AA: National Institute on Aging–Alzheimer's Association; NINCDS-ADRDA: National Institute of Neurological and Communicative Disorders and Stroke and the Alzheimer's Disease and Related Disorders Association; SPECT: single-photon emission computed tomography.

**Supplemental Table 3.** Additional characteristics of metabolism studies

| **Reference** | **Cognition screening instrument** | **Diagnosis criteria** | **Anosognosia measurement instrument** | **Method used to assess awareness** | **Note** |
| --- | --- | --- | --- | --- | --- |
| Harwood et al., 2005 | MMSE | NINCDS-ADRDA | Inaccurate Insight item of the Neurobehavioral rating scale | Discrepancy score between patient and informant interview and cognitive testing | No patients on AChEIs. |
| Salmon et al., 2006 | CDR | NINCDS-ADRDA and DSM-IV | Experimental questionnaire for the NEST-DD | Discrepancy score between patient and informant | 89 patients taking AChEIs , 39 taking antidepressants; 36 CDR score 0.5, 135 CDR score 1, 38 CDR score 2 |
| Nobili et al., 2010 | CDR | Petersen et al., 2004 | Memory Complaint Questionnaire | Self-accuracy discrepancy | CDR = 0.5; 14 progressed to AD in a period ranging from 6-28 months (mean 14.8±6.4), non-amnestic MCI excluded; unspecified number of patients taking SSRIs. |
| Jedidi et al., 2014 | Mattis dementia rating scale | NINCDS-ADRDA | Klein and colleagues’ personality traits questionnaire | Discrepancy score between patient and informant | Zarit's burden interview to the caregivers. |
| Sultzer et al., 2014 | Mattis dementia rating scale | NIA-AA | Inaccurate insight item of Neurobehavioral Rating Scale | Discrepancy score between patients’ assessment of his skills and the examiner’s overall assessment | 32 patients taking AChEIs, 21 patients taking SSRIs, none taking antipsychotics, benzodiazepine or other psychotropic medication. Analysis based on presence of delusions. |
| Gerretsen et al., 2017 | MMSE, MoCA and CDR | NINCDS-ADRDA | Everyday Cognition Scale | Discrepancy score between patient and informant | ADNI cohort. MCI defined as CDR=0.5 and MMSE 24 to 30; AD defined as MMSE≤26 and CDR≥0.5 |
| Therriault et al., 2018 | MMSE and CDR | Petersen et al., 2004 | Everyday Cognition Scale | Discrepancy score between patient and informant | ADNI cohort. MCI defined as CDR=0.5 and MMSE 24 to 30; AD defined as MMSE≤26 and CDR≥0.5 |

Abbreviations: AChEIs: acetylcholinesterase inhibitors; AD: Alzheimer’s Disease; ADNI: Alzheimer’s Disease Neuroimaging Initiative; CDR: Clinical Dementia Rating; DSM-IV: Diagnostic and Statistical Manual of Mental Disorders- IV; MCI: mild cognitive impairment; MMSE: Mini-mental state examination; MoCA: Montreal Cognitive Assessment;NIA-AA: National Institute on Aging–Alzheimer's Association; NINCDS-ADRDA: National Institute of Neurological and Communicative Disorders and Stroke and the Alzheimer's Disease and Related Disorders Association; SSRIs: selective serotonin reuptake inhibitors.

**Supplemental Table 4.** Additional characteristics of activation studies

| **Reference** | **Cognition screening instrument** | **Diagnosis criteria** | **Anosognosia measurement instrument** | **Method used to assess awareness** | **Note** |
| --- | --- | --- | --- | --- | --- |
| Ries et al., 2007 | MMSE | Winblad et al., 2004 | IQCODE | Discrepancy score between patient and informant | All 16 MCI patients taking AChEIs |
| Ruby et al., 2009 | CDR | NINCDS-ADRDA | Personality awareness score (Klein and colleagues’ personality traits questionnaire) | Discrepancy score between patient and informant | All subjects taking anti- AChEIs, a group with younger subjects was also analyzed. |
| Amanzio et al., 2011 | MMSE | NINCDS-ADRDA | AQ-D | Discrepancy score between patient and informant | Apathy and disinhibition among first behavioral changes in AD patients with anosognosia |
| Zamboni et al., 2013 | MMSE and MoCA | NINCDS-ADRDA and DSM-IV | AQ-D | Discrepancy score between patient and informant | No patients on AChEIs. Correlation between anosognosia and mPFC activation |

Abbreviations: AChEIs: acetylcholinesterase inhibitors; AD: Alzheimer’s disease; AQ-D: Anosognosia Questionnaire Dementia; CDR: Clinical Dementia Rating; DSM-IV: Diagnostic and Statistical Manual of Mental Disorders- IV; IQCODE: Informant Questionnaire on Cognitive Decline in the Elderly; MMSE: Mini-mental state examination; MCI: mild cognitive impairment; MoCA: Montreal Cognitive Assessment; mPFC: medial prefrontal cortex; NINCDS-ADRDA: National Institute of Neurological and Communicative Disorders and Stroke and the Alzheimer's disease and Related Disorders Association.

**Supplemental Table 5.** Additional characteristics of connectivity studies

| **Reference** | **Cognition screening instrument** | **Diagnosis criteria** | **Anosognosia measurement instrument** | **Method used to assess awareness** | **Note** |
| --- | --- | --- | --- | --- | --- |
| Ries et al., 2012 | CDR | NINCDS-ADRDA and Petersen et al., 2001 | Memory Awareness Rating Scale | Discrepancy score between patient and informant | ROI connectivity analysis; CDR=0.5-1 in AD/MCI group No separate within AD/MCI group analysis |
| Berlingeri et al., 2015 | MMSE | Not stated; only stated that previously diagnosed memory clinic patients included | AQ-D | Discrepancy score between patient and informant | ROI connectivity analysis |
| Perrotin et al., 2015 | MMSE | NINCDS-ADRDA | Self-Rating Scale of Memory Function | Delta score: Difference between subjective and objective episodic memory score | ROI connectivity analysis. Neuro-psychological test: Objective memory, subjective memory |
| Vannini et al., 2017 | MMSE and CDR | Petersen et al., 2004 | Memory Functioning Questionnaire | Delta score: Difference between subjective and objective episodic memory score | CDR=0.5 and MMSE 24 to 30; Hachinski score<4; ROI connectivity analysis based on FDG-PET hypometabolic seeds. |

Abbreviations: AD: Alzheimer’s disease; AQ-D: Anosognosia Questionnaire Dementia; CDR: Clinical Dementia Rating; MMSE: Mini-mental state examination; MCI: mild cognitive impairment; NINCDS-ADRDA: National Institute of Neurological and Communicative Disorders and Stroke and the Alzheimer's disease and Related Disorders Association; ROI: region of interest.

**Supplemental Table 6.** Search strategy for PubMed

|  | **Anosognosia** | **Alzheimer** | **Neuroimaging** |
| --- | --- | --- | --- |
| **MeSH** | “Anosognosia” [Mesh] OR "Diagnostic Self Evaluation" [Mesh] OR “Awareness” [Mesh] OR “Consciousness” [Mesh] OR “Metacognition” [Mesh] | "Alzheimer Disease" [Mesh] OR "Dementia" [Mesh] OR "Cognitive dysfunction" [Mesh] | “Neuroimaging” [Mesh] OR “Functional Neuroimaging” [Mesh] OR “Single Photon Emission Computed Tomography Computed Tomography” [Mesh] OR “Positron Emission Tomography” [Mesh] OR “Magnetic Resonance Imaging” [Mesh] |
| **Text words [tiab]** | Anosognosia* [tiab] OR insight [tiab] OR self-appraisal [tiab] OR self-conscious* [tiab] OR impaired [tiab] OR awareness [tiab] OR memory loss [tiab] | Alzheimer*[tiab] OR Mild Cognitive Impairment [tiab] OR MCI [tiab] OR dementia* [tiab] OR AD [tiab] OR aMCI [tiab] | MRI [tiab] OR PET [tiab] OR SPECT [tiab] OR fMRI [tiab] OR connectivity [tiab] OR activation [tiab] OR perfusion [tiab] OR metabolism [tiab] |
| (“Anosognosia” [Mesh] OR "Diagnostic Self Evaluation" [Mesh] OR “Awareness” [Mesh] OR “Consciousness” [Mesh] OR “Metacognition” [Mesh] OR Anosognosia* [tiab] OR insight [tiab] OR self-appraisal [tiab] OR self-conscious* [tiab] OR impaired [tiab] OR awareness [tiab] OR memory loss [tiab])  AND  ("Alzheimer Disease" [Mesh] OR "Dementia" [Mesh] OR "Cognitive dysfunction" [Mesh] OR Alzheimer* [tiab] OR Mild AND Cognitive AND Impairment [tiab] OR MCI [tiab] OR dementia* [tiab] OR AD [tiab] OR aMCI [tiab])  AND  (“Neuroimaging” [Mesh] OR “Functional Neuroimaging”[Mesh] OR “Single Photon Emission Computed Tomography” [Mesh] OR “Positron Emission Tomography”[Mesh] OR “Magnetic Resonance Imaging”[Mesh] OR MRI [tiab] OR PET [tiab] OR SPECT [tiab] OR fMRI [tiab] OR connectivity [tiab] OR activation [tiab] OR perfusion [tiab] OR metabolism [tiab]) | | | |
| 3516 titles 28-03-2018 | | | |

**Supplemental Table 7.** Search Strategy for PsycINFO

|  | **Anosognosia** | **Alzheimer** | **Neuroimaging** |
| --- | --- | --- | --- |
| DE | DE “Anosognosia” OR DE “Awareness” OR DE “Consciousness and cognition” OR DE “Metacognition” OR DE “memory loss” | DE "Alzheimer's disease” OR DE "Dementia" OR DE "Cognitive impairment" OR DE “Mild cognitive impairment” | DE “Neuroimaging” OR DE “Functional Neuroimaging” OR DE “Single Photon Emission Computed Tomography or SPECT” OR DE “Positron Emission Tomography or PET” OR DE “Magnetic Resonance Imaging or MRI” OR DE “fmri or functional magnetic resonance imaging” |
| Text words [tiab] | Insight OR self-appraisal OR self-consciousness OR impaired | MCI OR AD OR aMCI | connectivity OR activation OR perfusion OR metabolism |
| (DE “Anosognosia” OR DE “Awareness” OR DE “Consciousness and cognition” OR DE “Metacognition” OR DE “memory loss” OR Insight OR self-appraisal OR self-conscious* OR impaired)  AND  (DE "Alzheimer's disease” OR DE "Dementia" OR DE "Cognitive impairment" OR DE “Mild cognitive impairment” OR MCI OR AD OR aMCI)  AND  (DE “Neuroimaging” OR DE “Functional Neuroimaging” OR DE “Single Photon Emission Computed Tomography or SPECT” OR DE “Positron Emission Tomography or PET” OR DE “Magnetic Resonance Imaging or MRI” OR DE “fmri or functional magnetic resonance imaging” OR connectivity OR activation OR perfusion OR metabolism) | | | |
| 3614 titles 28-03-2018 | | | |

**Supplemental Table 8.** Search Strategy for EMBASE

| “Anosognosia” OR "Diagnostic Self Evaluation" OR “Awareness” OR “Consciousness” OR “Metacognition” OR Anosognosia* OR insight OR self-appraisal OR self-conscious* OR impaired OR awareness OR memory loss  AND  "Alzheimer Disease" OR "Dementia" OR "Cognitive dysfunction" OR Alzheimer* OR Mild AND Cognitive AND Impairment OR MCI OR dementia* OR AD OR aMCI  AND  “Neuroimaging” OR “Functional Neuroimaging” OR “Single Photon Emission Computed Tomography” OR “Positron Emission Tomography” OR “Magnetic Resonance Imaging” OR MRI OR PET OR SPECT OR fMRI OR connectivity OR activation OR perfusion OR metabolism |
| --- |
| 3564 titles 28-03-2018 |

**Supplemental Table 9.** Risk of bias in-depth assessment table

| **Reference** | **SPECT** |
| --- | --- |
| Reed et al., 1993 | Disease progression bias cannot be excluded although unlikely because of the time between neuropsychological evaluation and SPECT acquisition. |
| Starkstein et al., 1995 | Patients with an intermediate AQ-D score (15-31) were not included in the neuroimaging analysis, low risk of uncertain results bias is considered due to the focus of the research question. |
| Starkstein et al., 1996 | Low concern that the inclusion of ischemic vascular dementia (IVD) patients does not match the review question because the analysis of the neuroimaging assessment compares AD and IVD patients. |
| Derouesné et al., 1999 | Eight patients did not have SPECT but were screened for anosognosia and were included in the analysis of neuropsychiatric symptoms and unawareness of cognitive deficits, no bias though. |
| Vogel et al., 2005 | Uncertain results bias is a possibility, as 11 eligible patients were not included in the neuroimaging analysis; nonetheless, the probability is minor as the patients that underwent the SPECT study were randomly selected. Unspecified elapsed time between neuropsychological and neuroimaging assessment is a risk of introducing disease progression bias. |
| Mimura et al., 2006 | There is risk of recovery bias since 75% of AD patients were taking donepezil. Two different control groups, one for the imaging and the other for the recognition/awareness examination were used, this design increases the risk of diagnostic case-control bias as different selection process were used to sample patients with anosognosia and both control groups. |
| Hanyu et al., 2008 | Recovery bias possibly present as 12 AD patients included were under treatment with donepezil for an undisclosed amount of time. It is unlikely that disease progression bias exists, even though no mention of elapsed time is stated. |
| Shibata et al., 2008 | The risk of disease progression bias is present because the time interval between neuropsychological and neuroimaging was not specified. |
| Sedaghat et al., 2010 | The risk of disease progression bias is present because the time interval between neuropsychological and neuroimaging was not specified. |
| Tagai et al., 2018 | Low risk of expert sampling bias is present as the research question aims to study a population with a high probability of AD. Low risk for verification bias in this group of informants as they also underwent a thorough neuropsychological evaluation. |
| **PET** | |
| Harwood et al., 2005 | In this study, only the correlation between FDG metabolism and awareness of cognitive and functional deficits was explored; there was no analysis of between-group differences. There is no bias with the methodology of the patients included. |
| Salmon et al., 2006 | No risk of disease progression bias because the time interval between neuropsychological and neuroimaging was days apart, according to the authors. |
| Nobili et al., 2010 | Although an unspecified number of patients were taking selective serotonin reuptake inhibitors, the approach to address recovery bias was appropriate by tapering off and later discontinuing drugs known to depress brain synaptic transmissions, such as benzodiazepines and tricyclic antidepressants. |
| Jedidi et al., 2014 | Diagnostic case-control bias was avoided as the controls also underwent full anosognosia screening. The risk of verification bias is reduced by application of the Zarit's burden interview to the caregivers. No risk of disease progression bias because the time interval between neuropsychological and neuroimaging was days apart, according to the authors. |
| Sultzer et al., 2014 | Recovery bias is present as more than a third of the patients were under treatment with cholinesterase inhibitors. Verification bias is reduced as the assessment of insight incorporates a clinical interview, cognitive testing, and the caregiver´s report. |
| Gerretsen et al., 2017 | Everyday Cognition Scale is a measure of daily living and not a measure of self-awareness. Recall bias might be present. Verification bias is present since the informant did not undergo any neuropsychological assessment increasing the level of subjectivity of their responses about the psychological condition of the patient. |
| Therriault et al., 2018 | Everyday Cognition Scale is a measure of daily living and not a measure of self-awareness. Recall bias might be present. Verification bias is present since the informant did not undergo any neuropsychological assessment increasing the level of subjectivity of their responses about the psychological condition of the patient. |
| **fMRI activation** | |
| Ries et al., 2007 | Recovery bias could be present because MCI patients were taking cholinesterase inhibitors with stable dosages and because the time between neuropsychological assessment and neuroimaging was not specified; although very unlikely. |
| Ruby et al., 2009 | Self-judgement personality traits assessment correctly classifies anosognosia. |
| Amanzio et al., 2011 | Low risk for verification bias in this group of informants as they also underwent a thorough neuropsychological evaluation. |
| Zamboni et al., 2013 | Verification bias is present, as the informants did not undergo neuropsychological or clinical screening. |
| **fMRI connectivity** | |
| Ries et al., 2012 | Low risk for verification bias in this group of informants as they also underwent Zarit Burden Interview-Revised. |
| Berlingeri et al., 2015 | Withdrawal of 2 AD patients with anosognosia, 4 AD without anosognosia and 5 healthy controls. The risk of bias due to withdrawal affects test performance. Verification bias is present since the informant did not undergo any neuropsychological assessment increasing the level of subjectivity of their responses about the psychological condition of the patient. Disease progression bias cannot be discarded. |
| Perrotin et al., 2015 | Low risk of expert sampling bias is present as the research question aims to study a population with a high probability of AD. |
| Vannini et al., 2018 | Low risk of expert sampling bias is present as the research question aims to study a population with a high probability of developing AD. |

Abbreviations: AD: Alzheimer’s disease; AQ-D: Anosognosia Questionnaire Dementia; FDG: 18F fluorodeoxyglucose; fMRI: functional magnetic resonance imaging; IVD: ischemic vascular dementia; MCI: mild cognitive impairment; PET: positron emission tomography; SPECT: single-photon emission computed tomography.

**Supplemental Table 10.** Risk of bias individual item assessment

| **Individual quality item** | | **Question** | **Judgement** | **Type of bias** |
| --- | --- | --- | --- | --- |
| 1 | Patient spectrum | Is the spectrum of the patients included, representative of anosognosia patients? | Memory clinic recruited patients. | Limited challenge bias, diagnostic case-control bias |
| 2 | Reference standard | Does the reference standard target anosognosia correctly? | The diagnosis of anosognosia should include a questionnaire intended to assess awareness of memory deficit and clinical evaluation. Reference standard is clinical assessment by specialist. | Verification bias |
| 3 | Disease progression | Is the period between anosognosia evaluation and neuroimaging study short enough to be sure that no change in disease diagnosis occurred? | Anosognosia evaluation should be performed within 6 months’ period. More than 6 months could jeopardize the assurance that the population did not progress (e.g. aMCI to mild AD, mild AD to moderate AD or moderate AD to severe AD). | Disease progression bias, recovery bias |
| 4 | Partial verification | Did the whole sample receive verification using the anosognosia clinical assessment? | All patients need to undergo awareness of memory deficit testing. | Partial verification bias |
| 5 | Differential verification | Did the patients undergo assessment for anosognosia irrespective of imaging test result? | All patients undergoing assessment of anosognosia or awareness of memory deficit should undergo at least a clinical interview focused to diagnose anosognosia. | Differential verification bias |
| 6 | Incorporation | Is the anosognosia screening method independent of the neuroimaging study? | The neuroimaging study should not be part of the diagnosis assessment for the classification of anosognosia or impaired awareness of memory deficit. | Incorporation bias |
| 7 | Test review | Were the neuroimaging results interpreted without knowledge of the anosognosia classification results? | This type of blinded outcome assessment does not apply as the results from neuroimaging studies are not influenced by performance in the anosognosia psychometric evaluation or knowledge of anosognosia status. | Test review bias |
| 8 | Diagnostic review | Were the anosognosia classification results interpreted without knowledge of neuroimaging results? | Anosognosia or awareness classification should always be done before the analysis of neuroimaging results. | Diagnostic review bias |
| 9 | Clinical review | Were the same clinical data available when the test results were interpreted as would be available when the test is used in practice? | Pre-specified key clinical data criteria for anosognosia in dementia: patient’s stage of cognitive impairment, psychometric test results used to stage the patients, neuropsychological testing for differential diagnosis (e.g. NPI, HADS, etc.). | Clinical review bias |
| 10 | Uninterpretable results | Were uninterpretable/ intermediate test results reported? | Uninterpretable results should be reported if they exist, but should be excluded from the analysis of the results; if they are included in the analysis, it should be clearly stated how these results were handled. | Uncertain results bias |
| 11 | Withdrawals | Were withdrawals from the study explained? | All patients should be accounted for, stating the number of withdrawals and the reason, if known. | Bias due to withdrawal affecting test performance |

Abbreviations: AD: Alzheimer’s disease; aMCI: amnestic mild cognitive impairment; HADS: Hospital Anxiety and Depression Scale; NPI: Neuropsychiatric Inventory.

**Supplemental Table 11.** PRISMA Statement checklist of items

| **Section/Topic** | **Item #** | **Checklist Item** | **Reported on page #** |
| --- | --- | --- | --- |
| **Title** | 1 | Functional neural correlates of anosognosia in mild cognitive impairment and Alzheimer’s disease: a systematic review. | 1 |
| **Abstract** | 2 | Structured summary. | 1 |
| **Introduction** | | | |
| **Rationale** | 3 | Review in the context of what is already known. | 4-8 |
| **Objectives** | 4 | Explicit statements regarding primary and secondary objectives. | 6-7 |
| **Methods** | | | |
| **Protocol and registration** | 5 | Protocol not registered. | NA |
| **Eligibility criteria** | 6 | Study characteristics: inclusion and exclusion criteria stated. | 9-10 |
| **Information sources** | 7 | PubMed, EMBASE, and PsycINFO databases. | 9 |
| **Search** | 8 | Search strategy described. | 9-10 |
| **Study selection** | 9 | Process for selecting studies described. | 9-10 |
| **Data collection process** | 10 | Method of data extraction. | 10 |
| **Data items** | 11 | List and definition of all variables. | 10-11 |
| **Risk of bias in individual studies** | 12 | Description of method for risk of bias assessment of individual studies. | 11 |
| **Summary measures** | 13 | Functional neuroimaging classification stated. | 11 |
| **Synthesis of results** | 14 | Description of methods for handling data and combining results. | 10-11 |
| **Risk of bias across studies** | 15 | Description of assessment for external validity. | 11 |
| **Additional analysis** | 16 | No additional analysis performed. | NA |
| **Results** | | | |
| **Study selection** | 17 | Description of selection process. | 12 |
| **Study characteristics** | 18 | Description of data extracted. | 12-15 |
| **Risk of bias within studies** | 19 | Presentation of the risk of bias for each individual study. | 48-49 |
| **Results of individual studies** | 20 | Full outcomes considered for each study included. | 15-18 |
| **Synthesis of results** | 21 | No meta-analysis performed. | NA |
| **Risk of bias across studies** | 22 | Presented the risk of bias for the cumulative evidence. | 49-51 |
| **Additional analysis** | 23 | No additional analysis performed. | NA |
| **Discussion** | | | |
| **Summary of evidence** | 24 | Summary of evidence provided. | 19-23 |
| **Limitations** | 25 | General and specific limitations provided. | 26-27, 51-52 |
| **Conclusions** | 26 | General interpretation of the results provided. | 27-28 |
| **Funding** | 27 | Description of sources of funding. | 28 |

**Supplemental Figure 1.** Risk of bias methodological quality graph.

**
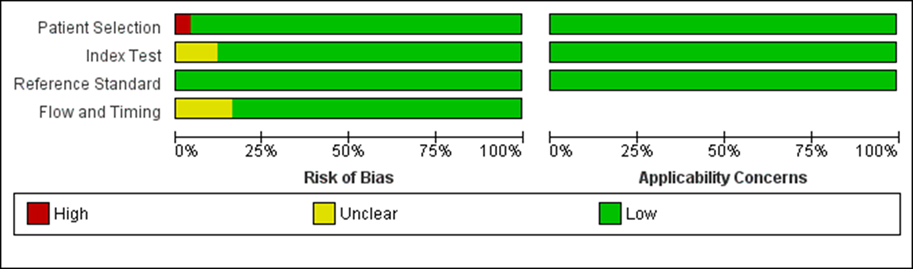
**

**Supplemental Figure 2.** Risk of bias methodological quality summary.

**
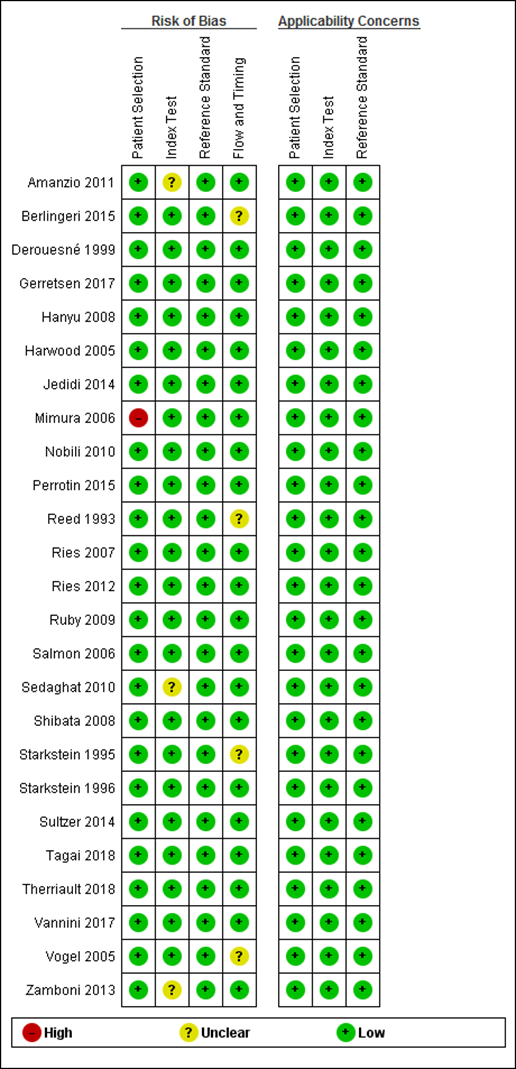
**
